# Inefficiencies and Patient Burdens in the Development of the Targeted Cancer Drug Sorafenib: A Systematic Review

**DOI:** 10.1371/journal.pbio.2000487

**Published:** 2017-02-03

**Authors:** James Mattina, Benjamin Carlisle, Yasmina Hachem, Dean Fergusson, Jonathan Kimmelman

**Affiliations:** 1 Studies of Translation, Ethics and Medicine (STREAM), Biomedical Ethics Unit, McGill University, Montréal, Quebec, Canada; 2 Department of Clinical Epidemiology, Ottawa Hospital Research Institute, Ottawa, Ontario, Canada; University of Sydney, Australia

## Abstract

Failure in cancer drug development exacts heavy burdens on patients and research systems. To investigate inefficiencies and burdens in targeted drug development in cancer, we conducted a systematic review of all prelicensure trials for the anticancer drug, sorafenib (Bayer/Onyx Pharmaceuticals). We searched Embase and MEDLINE databases on October 14, 2014, for prelicensure clinical trials testing sorafenib against cancers. We measured risk by serious adverse event rates, benefit by objective response rates and survival, and trial success by prespecified primary endpoint attainment with acceptable toxicity. The first two clinically useful applications of sorafenib were discovered in the first 2 efficacy trials, after five drug-related deaths (4.6% of 108 total) and 93 total patient-years of involvement (2.4% of 3,928 total). Thereafter, sorafenib was tested in 26 indications and 67 drug combinations, leading to one additional licensure. Drug developers tested 5 indications in over 5 trials each, comprising 56 drug-related deaths (51.8% of 108 total) and 1,155 patient-years (29.4% of 3,928 total) of burden in unsuccessful attempts to discover utility against these malignancies. Overall, 32 Phase II trials (26% of Phase II activity) were duplicative, lacked appropriate follow-up, or were uninformative because of accrual failure, constituting 1,773 patients (15.6% of 11,355 total) participating in prelicensure sorafenib trials. The clinical utility of sorafenib was established early in development, with low burden on patients and resources. However, these early successes were followed by rapid and exhaustive testing against various malignancies and combination regimens, leading to excess patient burden. Our evaluation of sorafenib development suggests many opportunities for reducing costs and unnecessary patient burden in cancer drug development.

## Author Summary

Numerous research subjects are exposed to unsafe and/or ineffective treatments in unsuccessful drug development programs. Yet, even successful drug development programs can involve heavy burdens for research subjects. In this manuscript, we measure risks and benefits for research subjects participating in the successful development of the anticancer drug sorafenib (first approved by the United States Food and Drug Administration in 2005). After discovering the first two cancer types responding to sorafenib, drug developers and researchers tested sorafenib against many other cancer types and in combination with many other drugs. We find that researchers were able to discover the utility of sorafenib for the first two cancer types quickly and with very little patient burden. Thereafter, attempts to extend the clinical application of sorafenib to other cancers and drug combinations involved many patients and adverse events and were mostly fruitless. We also find that many studies pursued after the first approval of sorafenib returned limited scientific information because they were duplicative or insufficiently informative. Our findings suggest that even successful drug development programs can entail substantial patient burden; they also point to ways that regulators, researchers, and policymakers can improve the risk-benefit ratio for research subjects.

## Introduction

In cancer, only 1 in 20 new drugs introduced to clinical development receives approval from the United States Food and Drug Administration (FDA) [1]. This high rate of attrition imposes burdens and opportunity costs on research subjects. It also consumes scarce human and material resources. Numerous studies have identified various sources of inefficiency in research, including poor priority setting, [2] biased study design, [3] underpowering, [4] and incomplete reporting [5]. Eliminating such inefficiencies holds promise for improving human protections and the social return on research investments.

Targeted therapies offer great promise for improving efficiencies and reducing burdens in cancer drug development. Indeed, targeted drugs like imatinib, sunitinib, or crizotinib have been approved for marketing on the basis of a small number of trials. Yet, little is known about total research activities and burdens for targeted drugs—especially those occurring after a drug receives its first regulatory approval. To quantify the patient burden and examine inefficiencies in cancer drug development, we undertook a systematic review of all published clinical trials for the drug sorafenib for which there was no FDA label at the time of trial launch (hereafter called “prelicensure trials”).

Sorafenib (Bayer/Onyx Pharmaceuticals) is the first multikinase inhibitor targeting RAF serine/threonine kinases and tumour vasculature [6]. Sorafenib was approved by the FDA for renal cell carcinoma (RCC) in 2005, [7] hepatocellular carcinoma (HCC) in 2007, [8] and radioactive iodine-refractory differentiated thyroid cancer in 2013 [9] and remains a current standard of care for each. In 2013, total sales were approximately €771 million [10]. Sorafenib has also frequently been used off-label, especially in patients whose tumours show alterations in relevant gene targets [11,12]. Our selection of sorafenib enabled us to capture trial activities for a novel targeted drug over 15 y, including almost a decade since initial FDA approval.

## Methods

### Literature Search

We searched Embase and MEDLINE databases on October 14, 2014, using the following search terms: “sorafenib” or “Nexavar” or variations of “BAY 43–9006,” and MeSH terms including variations of “clinical trial” or “randomized controlled trial” or other keywords associated with clinical trial design. No date restrictions were applied. Our complete search strategy can be found in the supplementary materials (see S1 Text).

### Study Eligibility

We applied the following inclusion criteria: (1) primary data, (2) full-text publication, (3) English language, (4) final report, (5) interventional trials, and (6) administered sorafenib as monotherapy or in a combination treatment regimen in (7) patients with a cancer diagnosis. We excluded articles that reported the following: (1) laboratory study of ex vivo human tissues, (2) case reports, (3) expansion cohorts of previously published trials, (4) adjuvant or maintenance therapy trials (these were excluded because the main measure of benefit used in our study, objective tumour response, is inapplicable), (5) expanded access, and (6) FDA on-label studies in which enrolment began after regulatory approval for the same indication and treatment regimen. For the purposes of this study, “on label” was defined on the basis of cancer site.

### Data Extraction

We extracted articles using a previously described template [13] that captures the following fields: (1) purpose and investigator conclusions, (2) study design and funding, (3) patient characteristics, (4) treatment procedures and duration, and (5) measures of patient risk and benefit—including response, survival, and adverse events. Trials were extracted independently by two coders using Numbat meta-analysis management software, [14] and disagreements were resolved by discussion. When data were not reported, we emailed corresponding authors. The response rate for missing data queries was 58% (19/33).

The duration of treatment was determined by the period between first drug exposure and the primary endpoint. For example, if a study used progression-free survival at 6 mo (PFS6) for the primary endpoint, duration of treatment was scored as 6 mo. For all other endpoints, we imputed duration of treatment by multiplying the median number of cycles administered by cycle length. The objective response rate (ORR) was defined as the proportion of confirmed complete and partial responses according to Response Evaluation Criteria In Solid Tumours (RECIST) or alternative response criteria if RECIST response was absent. We excluded haematological malignancies from all response analyses, since response evaluation criteria are noncomparable with solid tumours.

Treatment-related grade 3 to 5 adverse events (G3–5 AEs) according to the National Cancer Institute Common Terminology Criteria for Adverse Events (CTCAE) were captured. Safety reporting was highly variable from one trial to another. To standardize the safety data across trials, we captured the most frequent treatment-related grade 3–4 adverse event reported. Grade 5 events were captured separately. Safety data were expressed as a percentage of patients with a grade 3–5 adverse event who received at least one dose of sorafenib.

A trial was categorized as “positive” if the primary efficacy endpoint demonstrated a statistically significant positive effect and the authors reported acceptable toxicity. When primary endpoints were not declared, or if results were inconclusive according to prespecified criteria or a trial had multiple primary efficacy endpoints with conflicting outcomes, trials were scored as inconclusive. A trial was deemed “negative” if it failed to reach its primary endpoint with statistical significance (i.e., null result) or had an author assessment of unacceptable toxicity. For hybrid trials (e.g., Phase I–II trials), the primary endpoint was extracted and the trial outcome was scored from the higher phase component.

We deemed trials to have “limited value” when they were (1) duplicative Phase II, (2) not appropriately followed up, or (3) had recruitment failure. All criteria for limited value were specified before analysis. We considered a Phase II trial to be potentially duplicative if it matched a previous Phase II trial in its phase, patient characteristics (based on indication, histopathology/subtype, biomarker eligibility, number of prior therapies, types of prior therapies, response to prior therapies, and disease stage), and treatment regime (dose and combination drug). We restricted this to Phase II trials only, since these trials are aimed primarily at generating hypotheses that can be tested in more rigorous randomized trials, and they do not generally use clinical endpoints (hence, pooling of multiple small studies would not enable a more precise estimate of clinical utility). Our criteria for a trial that lacked follow-up included any Phase II trial that met its primary efficacy endpoint with a recommendation from the authors to pursue further testing that we were unable to identify a follow-up randomized trial using survival endpoints in the ClinicalTrials.gov or World Health Organization (WHO) clinical trial registries as of at least 3 y since trial close. We considered these trials to have limited value because they have delivered a signal of clinical promise but their hypotheses regarding clinical value remain unresolved and thus are inadequate for guiding practice. Recruitment failure was defined as any trial (Phase I through III) that failed to reach at least 85% of its targeted enrolment for reasons other than futility or benefit, based on trial report or registration. All trials were assessed independently by two coders for “limited value” status; raw agreement was 88%. All differences were resolved by discussion [15].

The above methods were published in a previous report [13]. However, the following methodological refinements were made in analysis stages because we believed they better captured the most relevant dynamics of efficiency and patient burden: (a) all figures involving time were presented by enrolment date rather than publication date, (b) criteria for studies of limited value were more stringent, (c) we included combination trials in our analysis, (d) we probed for trends in risk and benefit of trials using cumulative meta-analysis.

### Analysis and Statistics

Objective response rate and serious adverse event proportions with 95% confidence intervals were calculated with R version 3.2.3 [16]. Pooling of objective response rates and serious adverse event rates was done using the meta package for R, [17] using the DerSimonian and Laird random-effects method [18].

The differences in our analysis of risk (grade 3–5 adverse event rate) and benefit (objective response rate) between industry and nonindustry trial subgroups and between Phase I and Phase II trial subgroups were analysed in R version 3.2.3 [16] using a weighted regression analysis. We tested whether industry-funded studies had more favourable outcomes on the hypothesis that companies have more complete and timely access to preclinical and clinical evidence and that companies might be motivated to fund only those studies likely to produce a license. The individual trial results were weighted by the inverse variance of the rate estimate. For all inferential testing, we defined *p* < 0.05 to be statistically significant. As all inferential testing was exploratory, we did not adjust for statistical multiplicity.

To show how risks and benefits evolved during sorafenib development, we plotted cumulative rates of grade 3–5 adverse events and overall responses by trial phase. Cumulative effect estimates were calculated using the DerSimonian and Laird random-effects method.

To determine where burdens are most concentrated and to probe for research inefficiencies, we plotted Accumulation of Evidence and Research Organization (AERO) diagrams representing indication in the vertical axis and year of first patient enrolment in the horizontal. Each node represents a clinical trial categorized by trial phase (node shape) and primary endpoint attainment (node colour).

Survival data were collected from experiments with any nonsorafenib comparator arm in which survival endpoints included all patients. Hazard ratios were either reported in the study or were calculated from summary time-to-event data using formulas from Tierney et al [19].

### Patient Involvement

No patients were involved in setting the research question or the outcome measures, nor were they involved in the design or implementation of the study. Since we used data from previous clinical trials, we are unable to disseminate the results of the research to study participants directly.

## Results

### Study Characteristics

Our search captured 203 clinical trials, of which 74 administered sorafenib as monotherapy [20–93] and 132 tested sorafenib in combination with another anticancer therapy; [43,55,66,94–190] 3 trials tested sorafenib as both monotherapy and in a combination regimen [43,55,66]. As a whole, 11,355 patients were exposed to sorafenib in prelicensure trials in 26 malignancies, with an estimated 3,928 patient-years of involvement over a period of 13.2 y. A total of 4,988 patients received sorafenib as monotherapy, and 6,367 patients received sorafenib in combination therapy. The properties of trials in our sample are listed in Table 1, and a PRISMA flow diagram can be found in S1 Fig.

**Table 1:** Properties of extracted trials in our sample. Note that three trials [43,55,66] tested sorafenib as both monotherapy and in a combination regimen.

		Monotherapy trials [20–93] (k = 74)	Combination [43,55,66,94–222] (k = 132)
Phase	Phase I	13 (18%)	57 (43%)
	Phase I–II	1 (1%)	12 (9%)
	Phase II	56 (76%)	58 (44%)
	Phase III	4 (5%)	5 (4%)
Sponsor	Industry only	21 (28%)	56 (42%)
	Some nonindustry	42 (57%)	58 (44%)
	Not stated	11 (15%)	18 (14%)
Location of corresponding author	North America	43 (58%)	74 (56%)
	Europe	22 (30%)	41 (31%)
	Asia	9 (12%)	16 (12%)
	Africa	0 (0%)	1 (1%)
Number of centres	Single centre	16 (22%)	32 (24%)
	Multicentre	49 (66%)	74 (56%)
	Not stated	9 (12%)	26 (20%)
Randomized		13 (18%)	28 (21%)
Trials with a nonsorafenib comparator arm		9 (12%)	18 (14%)
Average duration of treatment (weeks)		15.3	17.2
Primary efficacy endpoint	Positive	16 (22%)	27 (20%)
	Inconclusive	11 (15%)	6 (5%)
	Negative	31 (42%)	45 (34%)
	Nonefficacy	16 (22%)	54 (41%)
Mean sample size	Efficacy	103	99
	Nonefficacy	49	26

### Patient Benefit and Burden of Sorafenib Exposure

In total, 1,486 patients receiving sorafenib experienced objective tumour response (12.1%, 95% CI 10.4% to 14.1%), and 108 died from drug-related toxicities (2.2%, 95% CI 1.9% to 2.5%). A minimum of 2,618 patients experienced grade 3–4 drug-related serious adverse events (24.1%, 95% CI 21.7% to 26.6%).

For monotherapy, 281 patients showed objective tumour response (6.8%, 95% CI 5.2% to 8.7%), and 23 patients died from drug-related toxicities (1.4%, 95% CI 1.1% to 1.8%); a minimum of 748 patients experienced grade 3–4 drug-related toxicities (15.3%, 95% CI 13% to 17.9%). In combination therapy, 1,205 patients experienced objective tumour response (17.0%, 95% CI 14.5% to 19.8%), and 85 patients died from treatment-related toxicity (2.6%, 95% CI 2.2% to 3.1%). A minimum of 1,870 patients experienced grade 3–4 drug-related toxicities (30.5%, 95% CI 27.2% to 34.0%).

### Clinical Development Trajectory

**Fig 1:**
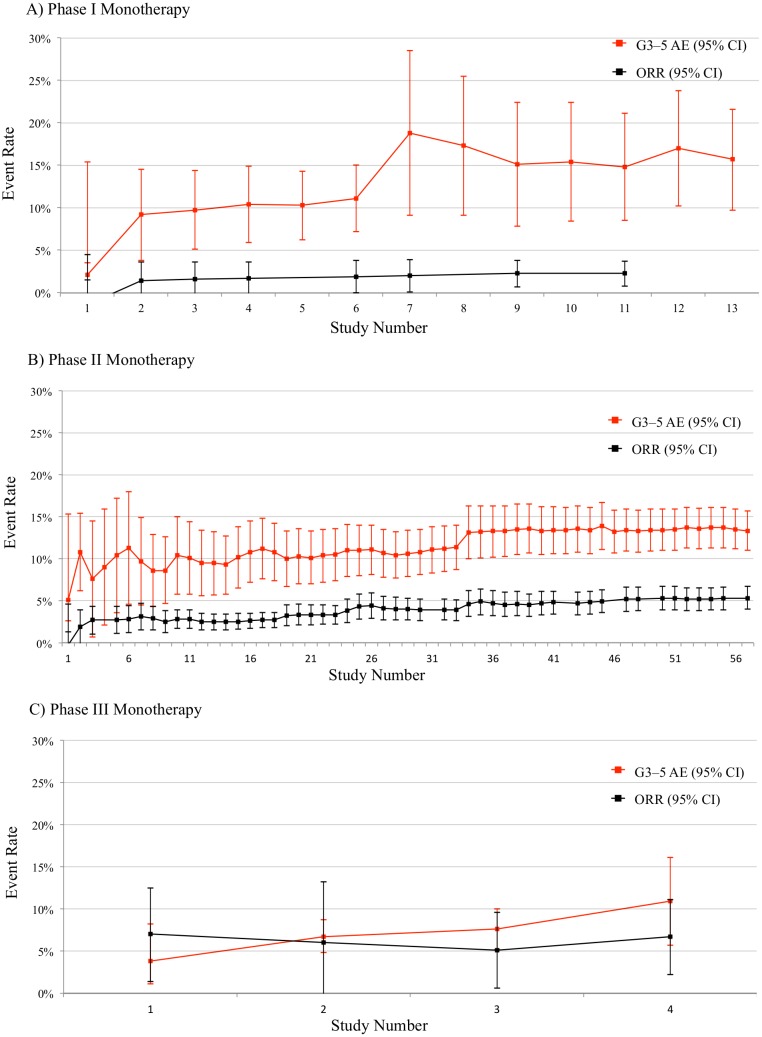
Cumulative risk (grade 3–5 serious adverse events [G3-5 AEs], in red) and benefit (overall response rate, in black) trends for sorafenib monotherapy. (A) Phase I, (B) Phase II, and (C) Phase III trials, ordered by enrolment date. Per-trial data are available in our supplementary materials (see S1 Data).

**Fig 2:**
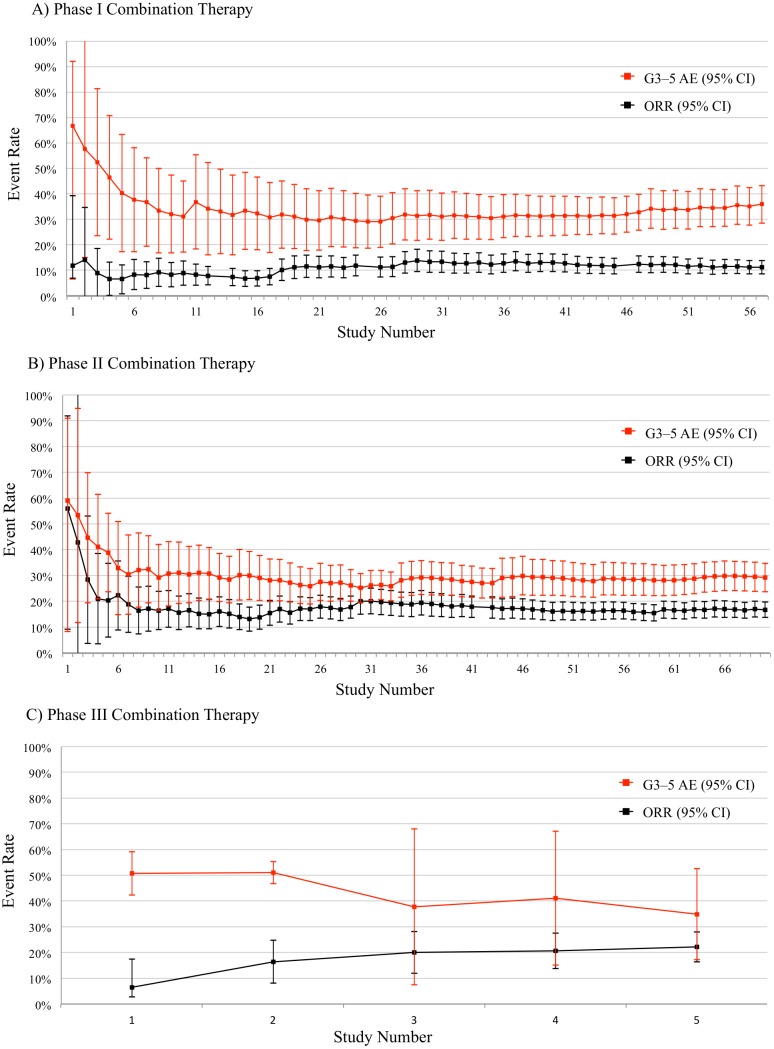
Cumulative risk (grade 3–5 serious adverse events [G3-5 AEs], in red) and benefit (overall response rate, in black) trends for sorafenib combination therapy. (A) Phase I, (B) Phase II, and (C) Phase III trials, ordered by enrolment date. Per-trial data are available in our supplementary materials (see S1 Data).

**Fig 3:**
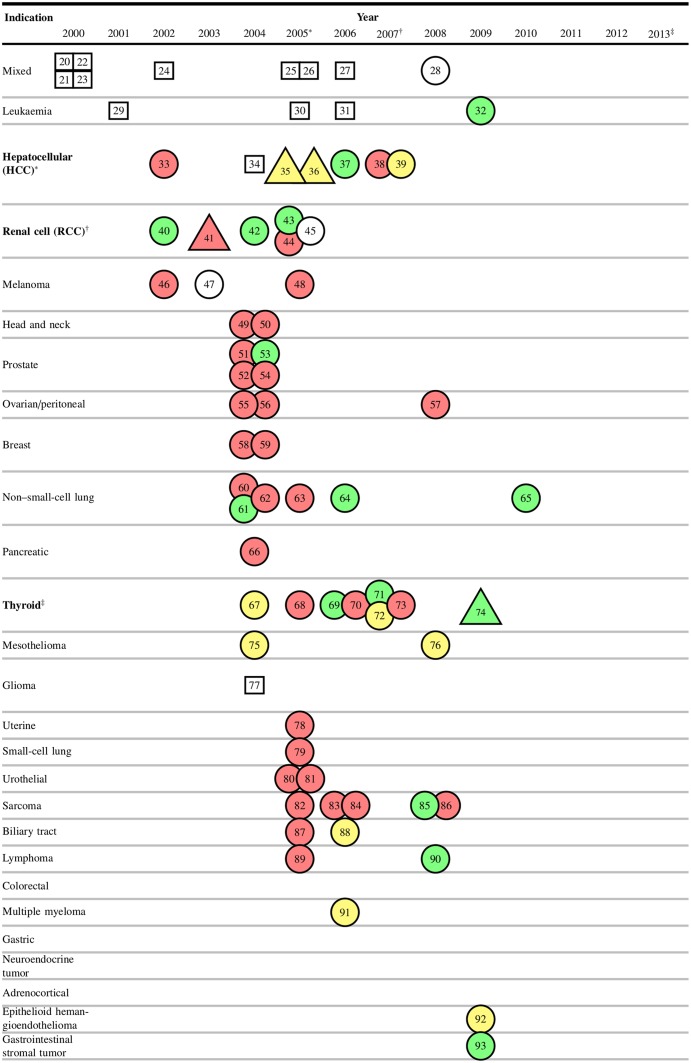
Accumulation of Evidence and Research Organization (AERO) diagram of sorafenib monotherapy trials, arranged by enrolment date. Rectangular nodes indicate Phase I trials, circular nodes indicate Phase II trials, and triangular nodes indicate Phase III trials. Green nodes indicate studies that reached their primary endpoint with acceptable toxicity (positive trials), yellow nodes indicate an inconclusive study, and red nodes indicate a failure to reach the primary endpoint or unacceptable toxicity (negative trials). White nodes are trials with nonefficacy primary endpoints. **Bold** denotes FDA-approved indications and corresponding years of licensure. Numbers in nodes represent the reference numbers of each trial. Per-trial data are available in our supplementary materials (see S1 Data).

**Fig 4:**
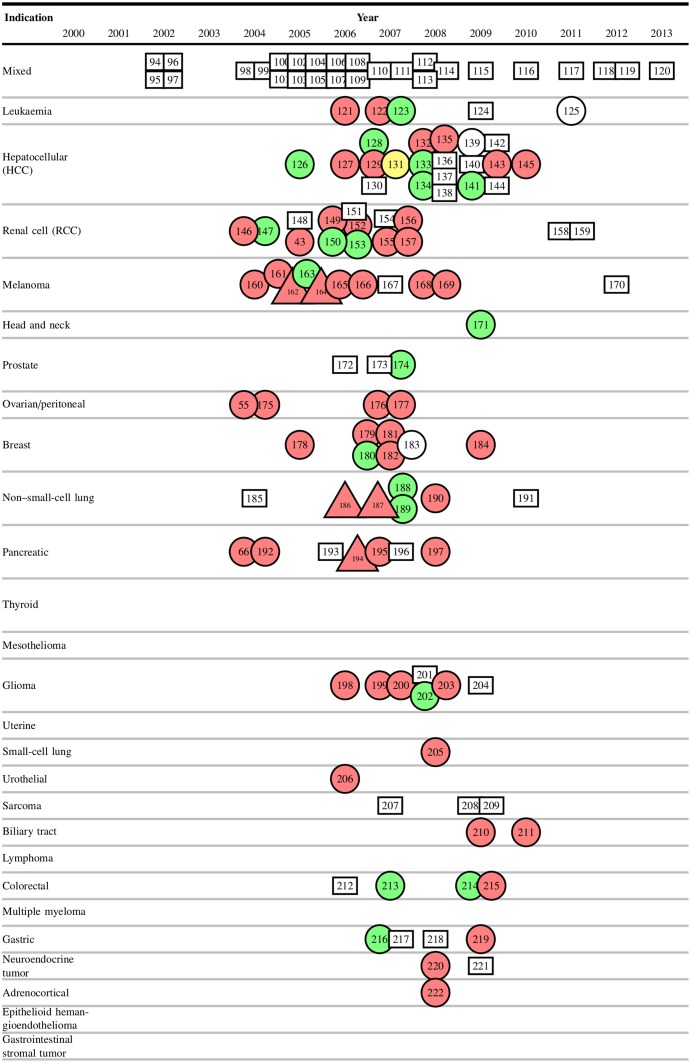
Accumulation of Evidence and Research Organization (AERO) diagram of sorafenib combination therapy trials, arranged by enrolment date. Rectangular nodes indicate Phase I trials, circular nodes indicate Phase II trials, and triangular nodes indicate Phase III trials. Green nodes indicate studies that reached their primary endpoint with acceptable toxicity (positive trials), yellow nodes indicate an inconclusive study, and red nodes indicate a failure to reach the primary endpoint or unacceptable toxicity (negative trials). White nodes are trials with nonefficacy primary endpoints. Numbers in nodes represent the reference numbers of each trial. Per-trial data are available in our supplementary materials (see S1 Data).

To show how risks and benefits evolved during sorafenib development, we plotted cumulative rates of grade 3–5 adverse events and objective responses for monotherapy (Fig 1) and combination therapy (Fig 2), by trial phase. Risk-benefit ratios remained relatively stable over the course of development in both monotherapy and combination therapy. We plotted AERO diagrams to show trial activities as a function of time and malignancy for monotherapy (Fig 3) and combination therapy (Fig 4). The apparent tapering of activities after year 2009 actually reflects that many trials now initiated have not yet published results and thus do not appear on the AERO diagram (on average, the interval between trial initiation and publication was 5 y). Optimal dose and schedule were identified in the earliest Phase I trials initiated in 2000 [20–23]. Initiation of two Phase II monotherapy trials occurred in 2002, each identifying an indication (renal cell carcinoma [40] and hepatocellular carcinoma [33]) that went on to receive licensure. At this time, sorafenib testing had accrued 5 drug-related deaths (4.6% of 108 total) and 93 total patient-years of involvement (2.4% of 3,928 total).

**Fig 5:**
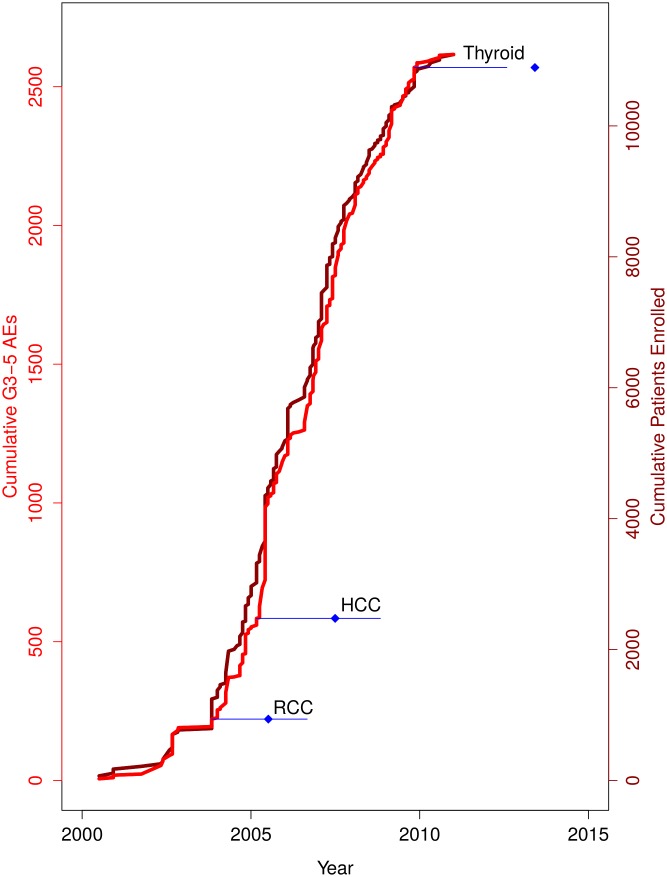
Cumulative treatment-related grade 3–5 adverse events (G3-5 AEs) (light red) and cumulative patients enrolled (dark red) in trials of sorafenib monotherapy and combination therapy over time with landmark events. Dates are based on first patient enrolment. Blue line segments indicate start and primary completion dates for pivotal trials in approved indications, and blue diamonds indicate new drug application (NDA) submissions leading to approval within 6 mo. RCC = renal cell carcinoma; HCC = hepatocellular carcinoma. Per-trial data are available in our supplementary materials (see S1 Data).

With the pivotal renal cell carcinoma trial underway, the exploration of new indications surged in 2004 and 2005—with 27 Phase II trials launched across 14 new indications (Figs 3 and 4)—and diminished thereafter. Two of these “exploratory” studies achieved statistical significance on their primary efficacy endpoint [53,61], one of which recommended further investigation of sorafenib monotherapy in non-small-cell lung carcinoma (NSCLC) [61]. From 2006 onwards, the majority of new trials investigated sorafenib in a combination regimen, with especially intensive investigation (>5 efficacy trials) of 5 indications: melanoma, ovarian cancer, breast cancer, non-small-cell lung carcinoma, and pancreatic cancer. Testing in these 5 indications comprised 56 drug-related deaths (51.8% of 108 total) and 1,155 patient-years (29.4% of 3,928 total) of burden in unsuccessful attempts to discover clinical utility. In total, sorafenib was tested in 26 indications and 67 drug combinations. Currently, sorafenib has not received an FDA label for any combination therapy, consistent with the unsuccessful Phase III trials in Fig 4. Fig 5 shows adverse events and number of patients enrolled as a function of time with landmark events.

The pivotal RCC Phase III trial [41] did not demonstrate a statistically significant advantage on its prespecified primary endpoint of overall survival (OS). This might have been due to early crossover; sorafenib was licensed on the basis of a significant progression-free survival advantage.

In HCC, the pivotal trial [35] identified coprimary endpoints of overall survival and time to symptomatic progression (TTSP). However, only overall median survival met the prespecified criteria for statistical significance, and the trial was represented as inconclusive with respect to the primary endpoint in Fig 3. The parallel Phase III study, designed for regulatory submissions in Asia [36], explicitly excluded a primary endpoint from the protocol and was also scored inconclusive on the AERO diagram (Fig 3). This study also reached statistical significance for overall survival but not time to symptomatic progression.

The most recent FDA approval occurred in November 2013 for metastatic differentiated thyroid cancer refractory to radioactive iodine treatment [9]. This was on the basis of a successful Phase III trial showing improved progression-free survival over placebo [74]; this trial did not demonstrate a statistically significant survival advantage.

**Fig 6:**
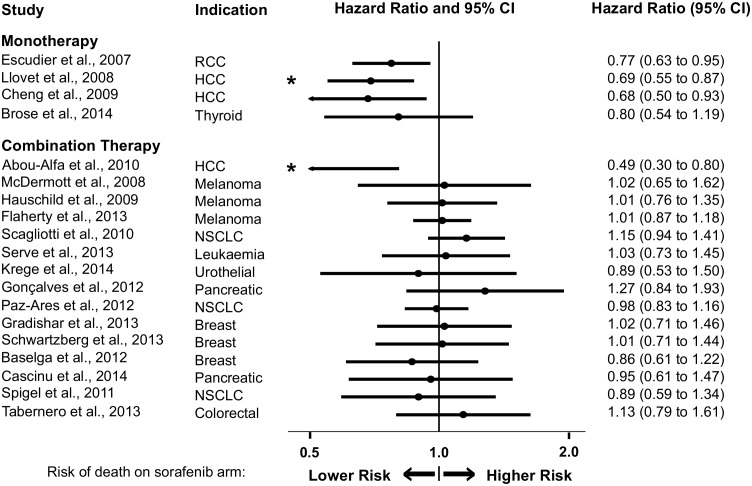
Hazard ratios (HRs) of overall median survival (OS) comparing monotherapy and combination therapy trials, arranged by trial initiation date (publication date shown). Asterisks indicate significant survival benefit according to prespecified statistical thresholds. RCC = renal cell carcinoma; HCC = hepatocellular carcinoma; NSCLC = non-small-cell lung carcinoma. Per-trial data are available in our supplementary materials (see S1 Data).

Analysis of overall survival revealed significant benefit against comparators in hepatocellular carcinoma in one monotherapy [35] and one combination trial [126] (Fig 6). For all other indications and combinations, exposure to sorafenib was neither demonstrably advantageous nor disadvantageous for patients, according to prespecified thresholds of significance. There was no obvious trend towards sorafenib disadvantage.

### Patterns of Research and Limited Value Studies

**Fig 7:**
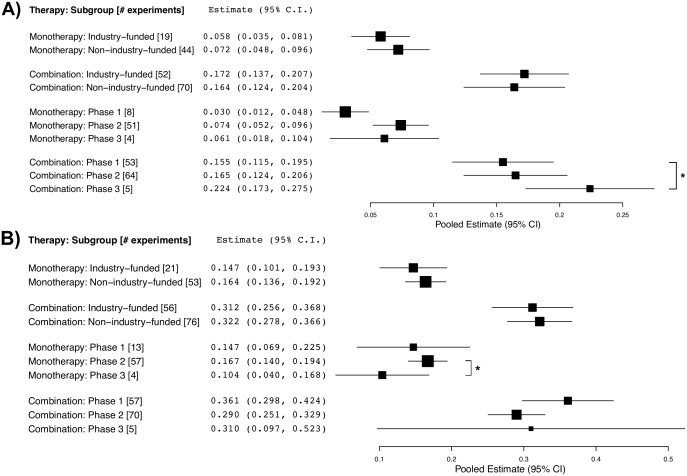
Pooled estimates and 95% CI for subgroup analyses. (A) objective response rate (ORR) and (B) grade 3–5 adverse events (G3–5 AEs) by therapy, funding source, and phase number. Asterisks indicate statistical significance (*p* < 0.05).

There was a significant improvement in objective response rate between Phase I and Phase III trials testing sorafenib in a combination regimen (15.5% and 22.4%, respectively; *p* < 0.05). Phase III trials testing sorafenib monotherapy reported a significantly lower grade 3–5 adverse event rate than Phase II trials (10.4% versus 16.7%, respectively; *p* < 0.05). All other analyses of objective response rate and grade 3–5 adverse events by funding source or trial phase were not significantly different between subgroups (Fig 7).

In total, 32 Phase II trials of limited value were identified (26% of Phase II trials), representing 1,773 patients and 701 y of patient involvement (15.6% and 17.8% of total prelicensure trial activity, respectively). In these trials, there were 367 grade 3–4 sorafenib-related adverse events and 9 patient deaths (14.0% of 2,618 adverse events and 8.3% of 108 deaths in sorafenib testing, respectively). Among the 1,717 patients in these studies evaluable for response in solid tumours, 269 achieved objective tumour response (18.1% of 1,486 responses in sorafenib testing).

Out of 124 Phase II studies, 10 were deemed potentially duplicative (8.1%) (S2 Table). In 14 instances, Phase II trials with a positive primary efficacy endpoint and author recommendation for further testing were not followed up in randomized trials testing survival (11.3%). Ten Phase II studies failed to accrue a sample sufficient to address the primary objectives of the study (8.1%; see S2 Text for details).

## Discussion

In this study, we extend our previous work exploring efficiency and burden in drug development. Consistent with our earlier reports looking at the anticancer drug sunitinib [13], we find that drug developers identified useful dosing, scheduling, and responding indications in a short amount of time with only a minimal number of trials, adverse events, or patient years. This suggests that drug developers can be highly effective at detecting signals of clinical promise using preclinical and preliminary clinical evidence—especially when there is a strong market incentive for efficiency.

However, once indications responding to sorafenib were identified, many indications and combination regimes were tested in rapid succession, leading to an accumulation of burden with greatly diminished return, as measured by FDA label revisions or confirmed clinical value. Cumulative risk and benefit analyses showed that in over a decade of drug development, the risk-benefit ratio remained relatively stable. This suggests that patients were not necessarily exposed to more unfavourable conditions with persistent testing but also that drug developers and clinical research groups were not able to mitigate risk or hone their ability to unlock greater clinical utility. Our analysis also found that patients allocated to sorafenib arms in randomized trials were generally neither advantaged nor disadvantaged in terms of survival (note that our pooled estimate may somewhat underestimate survival advantage because of crossover in HCC and RCC studies). This suggests that patient burdens entailed by this pattern of rapid-fire exploration are at least not associated with survival disadvantage for patients receiving sorafenib.

### Implications for Drug Developers

Our results also suggest that, while drug developers are adept at identifying and confirming correct hypotheses about clinical utility, researchers are slow to curtail attempts to discover new responding indications or to abandon hypotheses that flag in testing. Regarding the former, attempting to extend the label of a drug seems appropriate—especially where a drug has shown activity against two discrete malignancies. Elsewhere, we have argued that translation trajectories must contain at least some negative trials in order to furnish health care systems with adequate evidence [223]; not testing sorafenib against other plausible malignancies would have left oncologists uncertain about additional applications of the drug. However, our findings suggest that researchers were unable to join preclinical evidence together with a rapidly expanding clinical evidence base to extend the label of sorafenib. For a systematic review of preclinical evidence relating to monotherapy trials in this paper, see Mattina et al. 2016 [224]. It is possible that new adaptive trial designs might offer more efficient ways of ruling out nonresponding indications.

Reluctance to abandon hypotheses is illustrated by the highly perseverant and, until now, futile attempts to apply sorafenib to indications like non-small-cell lung carcinoma, breast cancer, and glioma. One potential explanation for such perseverance is clinical demand. Treatment of glioma has improved little in the last decade, and researchers might be willing to launch new glioma trials even if their confidence in promise is meagre. A second possibility is market demand: if the potential commercial returns are sufficiently large, drug companies may be willing to pursue trials that have marginal prospects of success. Third, seeming perseverance may reflect that many researchers test nearly identical hypotheses simultaneously, thus limiting the ability of researchers to build on insights. Favouring this interpretation is the fact that the periods of clinical testing for all trials deemed duplicative in our analysis overlapped. On the other hand, many trial activities for highly tested indications such as breast cancer did not overlap in terms of time period. Last, researchers may be prone to cognitive biases by which evidence confirming a molecular hypothesis is given greater weight than evidence disconfirming it. Sorafenib was tested against melanoma, generally on the conviction that sorafenib can interrupt disease progression through the MAPK pathway. However, seven trials that were founded on this premise had negative outcomes, resulting in potentially excess exposures [46,48,160,161,165,168,169]. The pattern of testing we observed is consistent with a dynamic in which researchers embrace findings when they support a pathophysiological premise but doubt their veracity when results conflict with favoured pathophysiological premises. The possible effects of cognitive biases on clinical development decisions deserves further exploration.

Our analysis raises a natural question: at what point should researchers discontinue attempts to extend a drug’s label to other indications and drug combinations? Reasonable people might disagree. The fact that cancer is a life-threatening illness would favour the kind of exhaustive testing we observed, as would the prospect that a novel multikinase inhibitor like sorafenib might show activity against a variety of malignancies. Under conditions of high uncertainty, it could make sense to launch many “hypothesis-generating” Phase II trials [225]. In our view, however, several points would caution against the extensive and perseverant testing we observed after licensure. First, the nontrivial toxicities associated with sorafenib—especially when combined with other drugs—would demand grounding any new trial in a good evidentiary rationale. Second, the fact that sorafenib is a targeted drug and that drug developers were so adept at selecting viable hypotheses early on should favour a higher proportion of successes in extending the label of sorafenib. Third, further testing of a drug ties up human and material resources that might be applied to other research programs. In a realm like cancer, where there is a dense pipeline of novel drug candidates and pressing clinical need, these opportunity costs should encourage judicious launch of new trials [225].

Our findings identify other opportunities to decrease patient burden and improve research efficiency. According to the criteria we used, 26% of prelicensure Phase II trials of sorafenib had limited value because they were duplicative, not followed up as appropriate, or unable to recruit a critical mass of subjects. To be sure, that a study is destined to have “limited value” is not always knowable at trial outset. Yet, knowing the volume of studies that—in retrospect—had limited value can motivate development of mechanisms and practices that reduce their occurrence. For instance, duplicative trials were always conducted during overlapping time periods; researchers, ethics committees, and data safety monitoring bodies should monitor trial registries for identical studies that have already started recruitment, and reevaluate risk and benefit when there is duplication. Others have observed that many positive, exploratory studies are not followed up with rigorous testing—particularly in combination trials [226]. It may seem counterintuitive to label any “positive” trial as having “limited value.” However, a large percentage of drug prescriptions in cancer are off-label, [227,228] and in numerous cases, positive surrogate responses that have not been promptly followed up with rigorous testing have led to adoption of treatments that were later shown ineffective and harmful [229–231]. Less duplication and better follow up of promising findings might be achieved by improving the flow of information and coordination among research teams. Trials with unsuccessful accrual are less likely to meet the statistical power needed to answer their primary question and impose unnecessary burden on patients and resources in the process. Ethics committees, investigators, and data safety monitoring bodies should closely monitor the design, recruitment, and feasibility of meeting accrual goals throughout a trial to mitigate risks from slow accrual [15].

### Study Limitations

Our findings should be interpreted in light of the following limitations. First, this analysis focused solely on published trial reports and is thus susceptible to the effects of publication bias. Because of labour limitations, for example, we did not search clinical trial registries for completed but unpublished studies. However, incorporating unpublished studies would not be expected to improve the favourability of risk-benefit ratios or to alter the temporal dynamics we observed. Second, this analysis used objective response rate in solid tumours as a proxy for benefit and treatment-related serious adverse events as a proxy for patient burden. We used response because it allows comparison of benefit across solid tumours and trial phases. However, response is a surrogate endpoint. Moreover, sorafenib is cytostatic in many malignancies, and clinical benefit may not be reflected in objective response rate. Treatment-related adverse events were also inconsistently reported—many studies only reported adverse events occurring in over a certain percentage of patients. Our measure of risk likely underestimates the real estimates. Third, some might question our criteria for “limited value” trials. Our definition of duplication probably excluded trials that many would consider duplicative or overlapping or that a meta-analyst would consider similar enough to aggregate. Indeed, several studies we classified as distinct using our criteria would almost certainly be viewed by many oncologists as substantially overlapping if not identical (see S2 Text). On the other hand, there may be grounds for believing some studies we classified as duplicative are distinct because they enrolled different demographic groups. As well, we note that even studies deemed to have “limited value” yield some information. For example, studies that are underpowered because of recruitment failure might be combined with similar studies in a meta-analysis. However, this places the onus of deriving scientific insights on meta-analyses that might or might not materialize; the important question is whether the value of this information is sufficient to purchase their patient burden and opportunity costs. Also, studies that have “limited value” might sometimes be beyond the control of sponsors or research teams. If the standard of care shifts radically, studies might close for low recruitment or positive Phase II studies might not have follow-up. It is therefore impossible to exclude that some studies classified as having limited value reflect dynamism elsewhere in cancer drug development. Last, the ethical implications of our findings need to be interpreted against the fact that the vast majority of trials in our sample enrolled patients with refractory disease. Nothing in our analysis would suggest such patients were deprived of standard of care or that enrolment was associated with survival disadvantage. Nevertheless, even when patients have advanced disease, the imposition of burdens like side effects and time commitment should be grounded in a compelling biological rationale.

## Conclusions

Our findings—when interpreted alongside the other drug development trajectory we analysed—suggest that drug developers can marshal preclinical and early trial evidence to discover a drug’s utility very efficiently. Inefficiencies, costs, and burdens accumulate later in drug development, and the majority of patient burden—however measured—accumulates after successful interventional strategies have been discovered. The patterns we observe suggest that a combination of commercial considerations, poor coordination, time pressures, and cognitive biases may distort trial decision making, resulting in substantial excess burdens and resource demands.

## Supporting Information

S1 Fig PRISMA flow diagram. (TIFF)

S2 Fig Forest plot of objective response rates (ORR) per trial, by funding source, for monotherapy trials. There was no significant difference in objective response rate (p > 0.05) between trials with industry-only funding and trials with at least one non-industry funding source.
(PDF)

S3 Fig Forest plot of treatment-related grade 3–5 adverse events per trial, by funding source, for monotherapy trials. There was no significant difference in grade 3–5 serious adverse event rate (p > 0.05) between trials with industry-only funding and trials with at least one non-industry funding source.
(PDF)

S4 Fig Forest plot of objective response rates (ORR) per trial, by phase, for monotherapy trials. There was no significant difference in objective response rate (p > 0.05) between phase I, phase II and phase III trials.
(PDF)

S5 Fig Forest plot of treatment-related grade 3–5 adverse events per trial, by phase, for monotherapy trials. There was a significant improvement in grade 3–5 serious adverse event rate (p < 0.05) between phase II and phase III trials. No other comparisons showed significance (p > 0.05).
(PDF)

S6 Fig Forest plot of objective response rates (ORR) per trial, by funding source, for combination therapy trials. There was no significant difference in objective response rate (p > 0.05) between trials with industry-only funding and trials with at least one non-industry funding source.
(PDF)

S7 Fig Forest plot of treatment-related grade 3–5 adverse events per trial, by funding source, for combination therapy trials. There was no significant difference in grade 3–5 serious adverse event rate (p > 0.05) between trials with industry-only funding and trials with at least one non-industry funding source.
(PDF)

S8 Fig Forest plot of objective response rates (ORR) per trial, by phase, for combination therapy trials. There was a significant improvement in objective response rate (p < 0.05) between phase I and phase III trials. No other comparisons showed significance (p > 0.05).
(PDF)

S9 Fig Forest plot of treatment-related grade 3–5 adverse events per trial, by phase, for combination therapy trials. There was no significant difference in grade 3–5 serious adverse event rate (p > 0.05) between phase I, phase II and phase III trials.
(PDF)

S1 Table PRISMA research checklist. (DOC)

S2 Table Substantially overlapping trials by eligibility criteria. (DOCX)

S1 Text Embase and MEDLINE search strategy. (DOCX)

S2 Text Citations of limited value trials by prespecified criteria. (DOCX)

S3 Text Research protocol adapted from a previous report [13]. (DOCX)

S4 Text Codebook for extraction of information from clinical trials. (DOCX)

S1 Data Dataset of clinical trials. (XLSX)

## Abbreviations

AERO: Accumulation of Evidence and Research Organization
HCC: hepatocellular carcinoma
NSCLC: non-small-cell lung carcinoma
ORR: objective response rate
OS: overall survival
RCC: renal cell carcinoma
TTSP: time to symptomatic progression
