# Supplementary figures and images for: Inefficiencies and Patient Burdens in the Development of the Targeted Cancer Drug Sorafenib: A Systematic Review
Source: PLoS Biol. 2017 Feb 3;15(2):e2000487. doi: 10.1371/journal.pbio.2000487 (PMC5291369; doi:10.1371/journal.pbio.2000487)

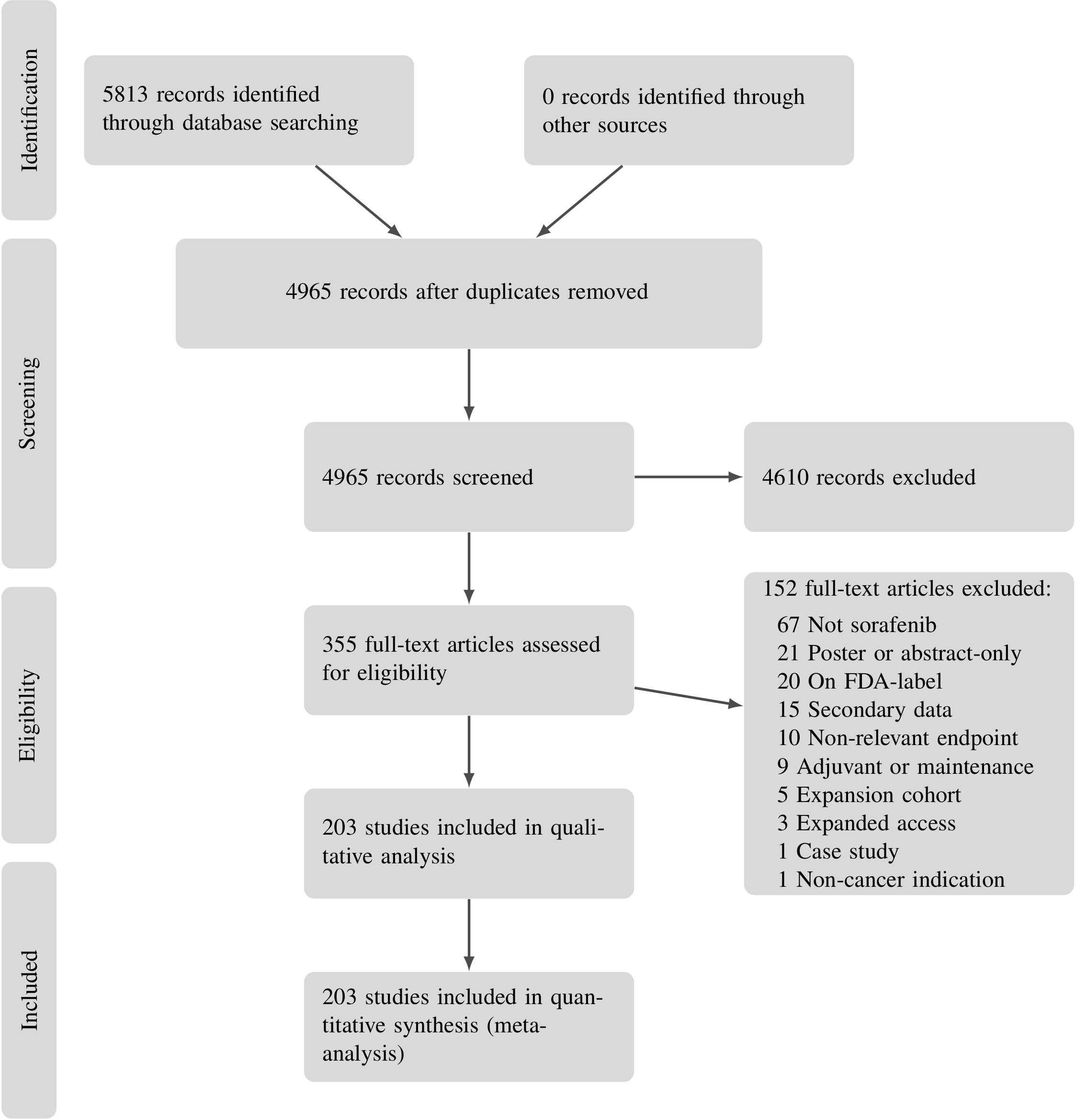

Supplement: S1 Fig — (TIFF) [file pbio.2000487.s001.tiff]

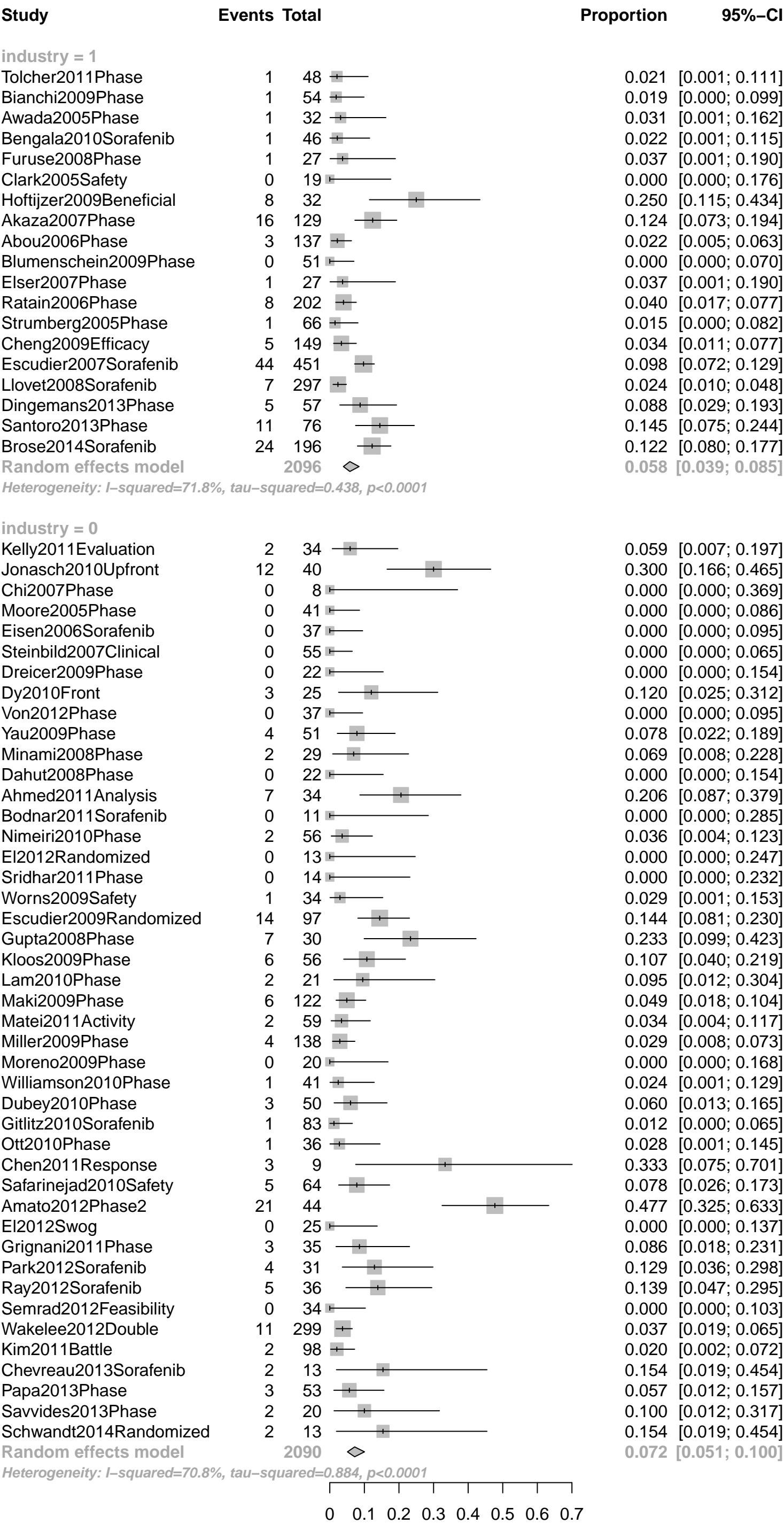

Supplement: S2 Fig — There was no significant difference in objective response rate (p > 0.05) between trials with industry-only funding and trials with at least one non-industry funding source. (PDF) [file pbio.2000487.s002.pdf]

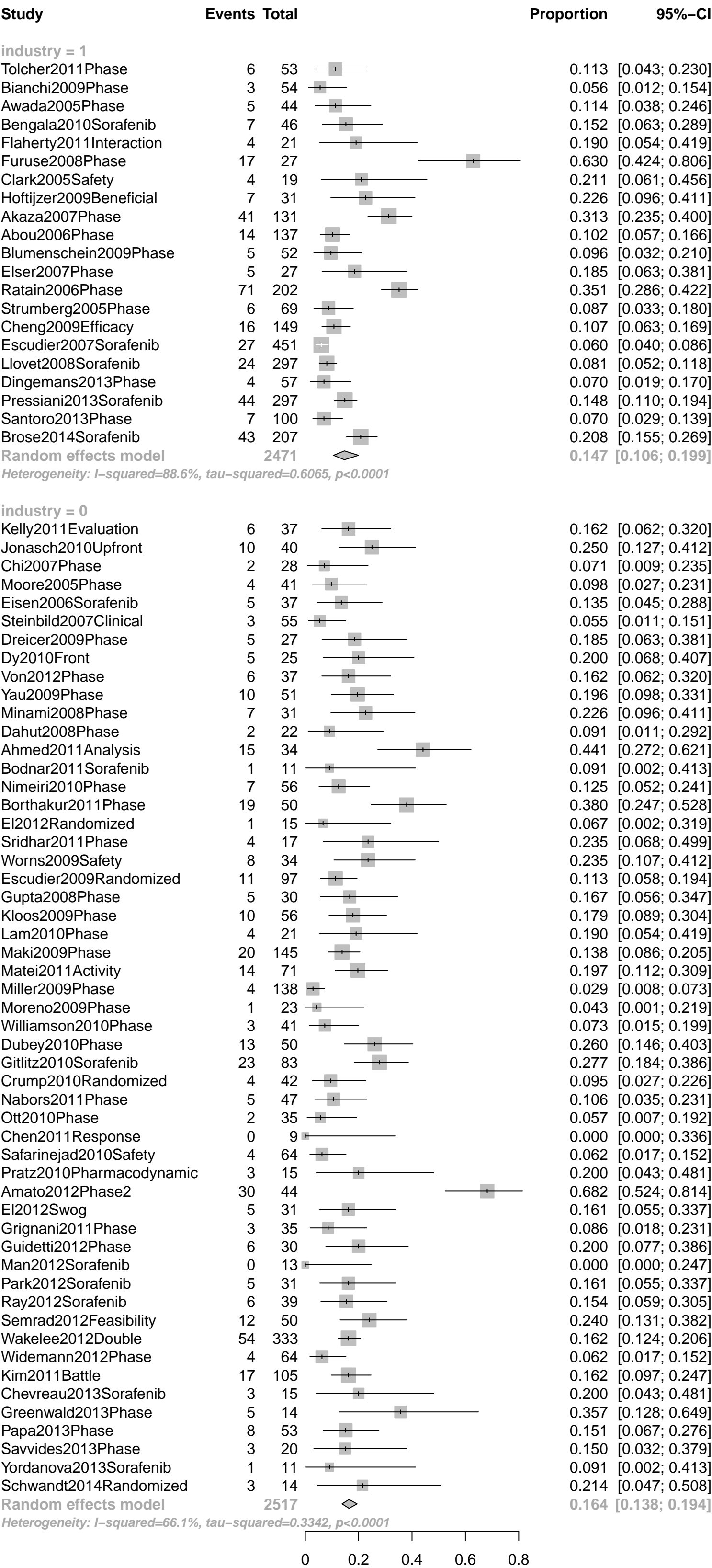

Supplement: S3 Fig — There was no significant difference in grade 3–5 serious adverse event rate (p > 0.05) between trials with industry-only funding and trials with at least one non-industry funding source. (PDF) [file pbio.2000487.s003.pdf]

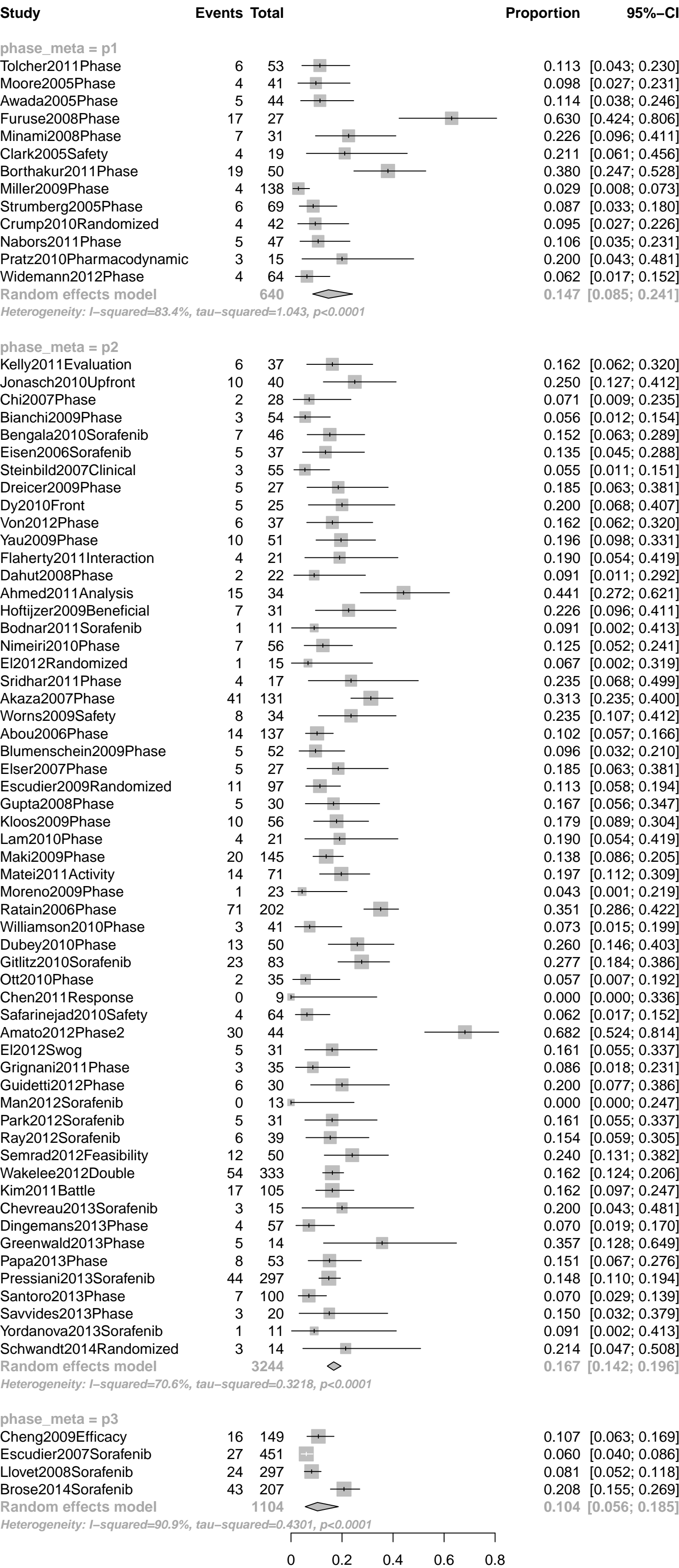

Supplement: S5 Fig — There was a significant improvement in grade 3–5 serious adverse event rate (p < 0.05) between phase II and phase III trials. No other comparisons showed significance (p > 0.05). (PDF) [file pbio.2000487.s005.pdf]

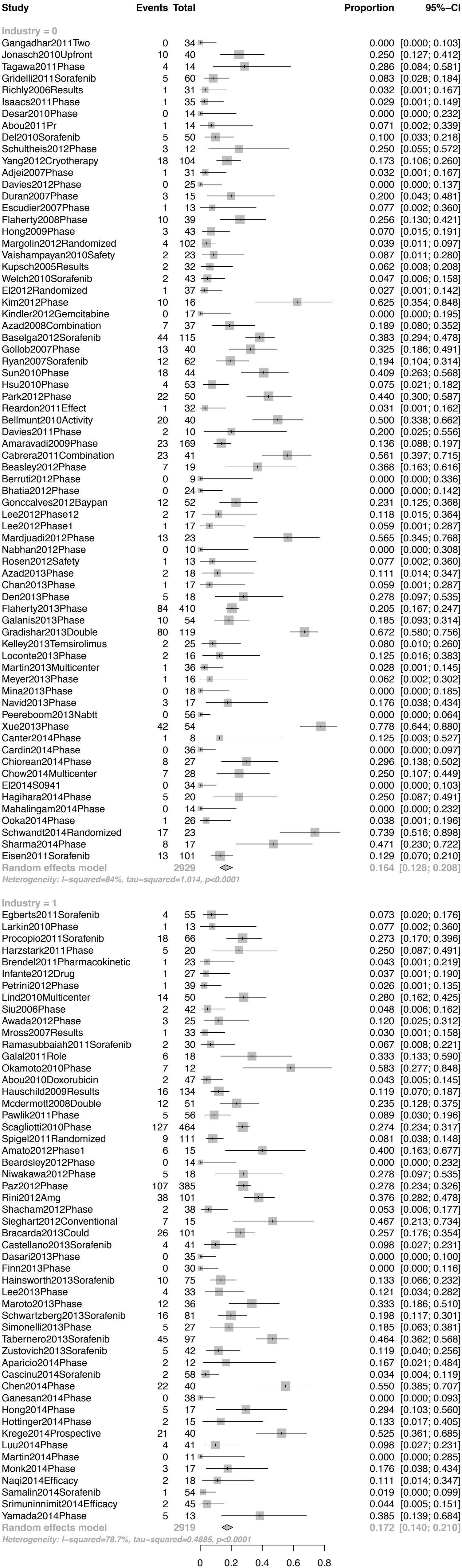

Supplement: S6 Fig — There was no significant difference in objective response rate (p > 0.05) between trials with industry-only funding and trials with at least one non-industry funding source. (PDF) [file pbio.2000487.s006.pdf]

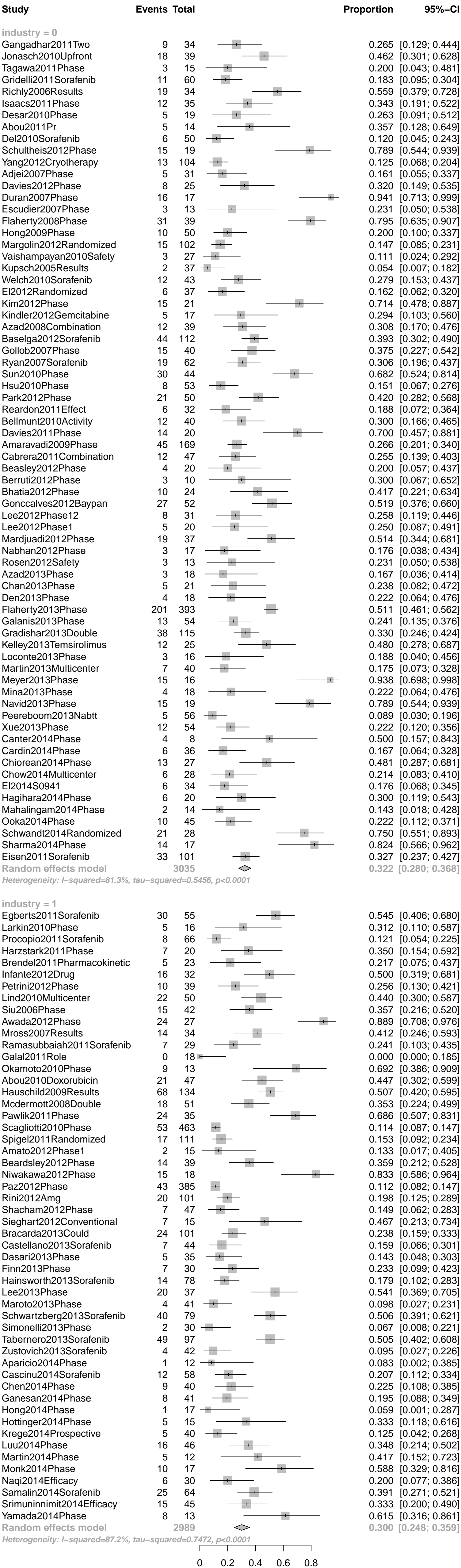

Supplement: S7 Fig — There was no significant difference in grade 3–5 serious adverse event rate (p > 0.05) between trials with industry-only funding and trials with at least one non-industry funding source. (PDF) [file pbio.2000487.s007.pdf]

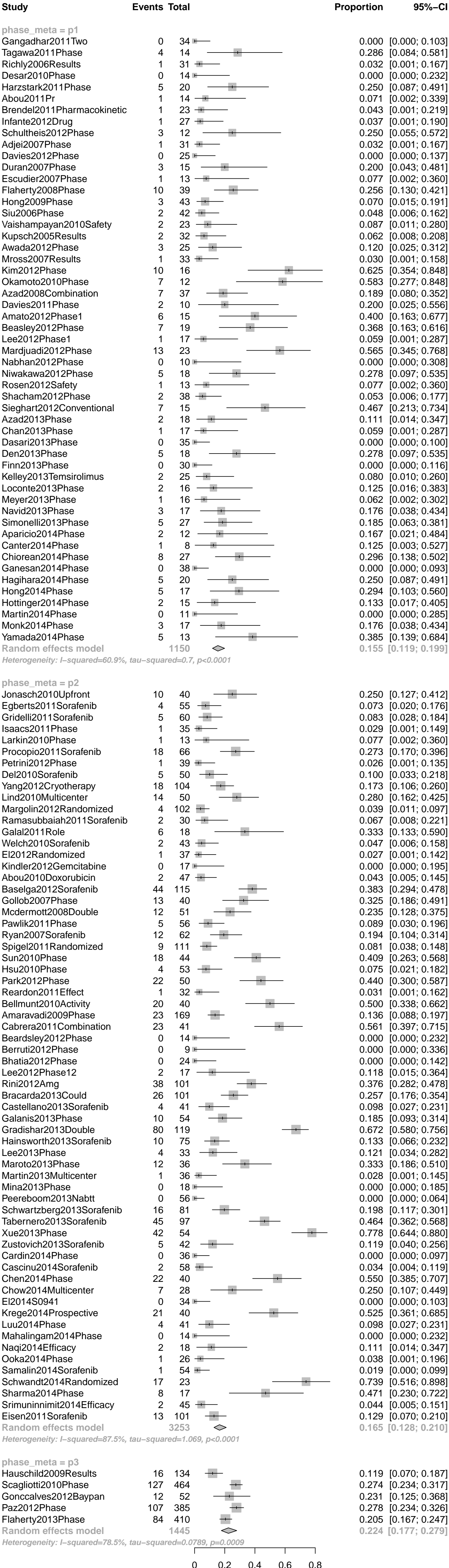

Supplement: S8 Fig — There was a significant improvement in objective response rate (p < 0.05) between phase I and phase III trials. No other comparisons showed significance (p > 0.05). (PDF) [file pbio.2000487.s008.pdf]

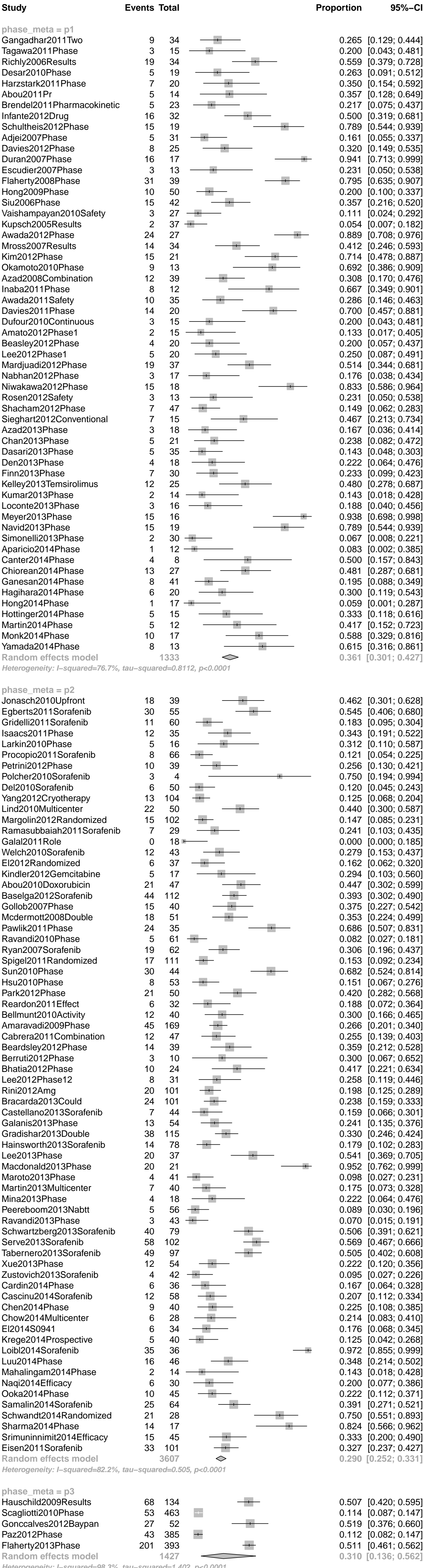

Supplement: S9 Fig — There was no significant difference in grade 3–5 serious adverse event rate (p > 0.05) between phase I, phase II and phase III trials. (PDF) [file pbio.2000487.s009.pdf]
