# Supplementary material for: Inefficiencies and Patient Burdens in the Development of the Targeted Cancer Drug Sorafenib: A Systematic Review
Source: PLoS Biol. 2017 Feb 3;15(2):e2000487. doi: 10.1371/journal.pbio.2000487 (PMC5291369; doi:10.1371/journal.pbio.2000487)

Study	Events	Total	Proportion	95%−CI
phase_meta = p1				
Tolcher2011Phase	1	48	0.021	[0.001; 0.111]
Moore2005Phase	0	41	0.000	[0.000; 0.086]
Awada2005Phase	1	32	0.031	[0.001; 0.162]
Furuse2008Phase	1	27	0.037	[0.001; 0.190]
Minami2008Phase	2	29	0.069	[0.008; 0.228]
Clark2005Safety	0	19	0.000	[0.000; 0.176]
Miller2009Phase	4	138	0.029	[0.008; 0.073]
Strumberg2005Phase	1	66	0.015	[0.000; 0.082]
**Random effects model**		**400**	**0.030**	**[0.017; 0.054]**
Heterogeneity: I−squared=0%, tau−squared=0, p=0.9237				
phase_meta = p2				
Kelly2011Evaluation	2	34	0.059	[0.007; 0.197]
Jonasch2010Upfront	12	40	0.300	[0.166; 0.465]
Chi2007Phase	0	8	0.000	[0.000; 0.369]
Bianchi2009Phase	1	54	0.019	[0.000; 0.099]
Bengala2010Sorafenib	1	46	0.022	[0.001; 0.115]
Eisen2006Sorafenib	0	37	0.000	[0.000; 0.095]
Steinbild2007Clinical	0	55	0.000	[0.000; 0.065]
Dreicer2009Phase	0	22	0.000	[0.000; 0.154]
Dy2010Front	3	25	0.120	[0.025; 0.312]
Von2012Phase	0	37	0.000	[0.000; 0.095]
Yau2009Phase	4	51	0.078	[0.022; 0.189]
Dahut2008Phase	0	22	0.000	[0.000; 0.154]
Ahmed2011Analysis	7	34	0.206	[0.087; 0.379]
Hoftijzer2009Beneficial	8	32	0.250	[0.115; 0.434]
Bodnar2011Sorafenib	0	11	0.000	[0.000; 0.285]
Nimeiri2010Phase	2	56	0.036	[0.004; 0.123]
El2012Randomized	0	13	0.000	[0.000; 0.247]
Sridhar2011Phase	0	14	0.000	[0.000; 0.232]
Akaza2007Phase	16	129	0.124	[0.073; 0.194]
Worns2009Safety	1	34	0.029	[0.001; 0.153]
Abou2006Phase	3	137	0.022	[0.005; 0.063]
Blumenschein2009Phase	0	51	0.000	[0.000; 0.070]
Elser2007Phase	1	27	0.037	[0.001; 0.190]
Escudier2009Randomized	14	97	0.144	[0.081; 0.230]
Gupta2008Phase	7	30	0.233	[0.099; 0.423]
Kloos2009Phase	6	56	0.107	[0.040; 0.219]
Lam2010Phase	2	21	0.095	[0.012; 0.304]
Maki2009Phase	6	122	0.049	[0.018; 0.104]
Matei2011Activity	2	59	0.034	[0.004; 0.117]
Moreno2009Phase	0	20	0.000	[0.000; 0.168]
Ratain2006Phase	8	202	0.040	[0.017; 0.077]
Williamson2010Phase	1	41	0.024	[0.001; 0.129]
Dubey2010Phase	3	50	0.060	[0.013; 0.165]
Gitlitz2010Sorafenib	1	83	0.012	[0.000; 0.065]
Ott2010Phase	1	36	0.028	[0.001; 0.145]
Chen2011Response	3	9	0.333	[0.075; 0.701]
Safarinejad2010Safety	5	64	0.078	[0.026; 0.173]
Amato2012Phase2	21	44	0.477	[0.325; 0.633]
El2012Swog	0	25	0.000	[0.000; 0.137]
Grignani2011Phase	3	35	0.086	[0.018; 0.231]
Park2012Sorafenib	4	31	0.129	[0.036; 0.298]
Ray2012Sorafenib	5	36	0.139	[0.047; 0.295]
Semrad2012Feasibility	0	34	0.000	[0.000; 0.103]
Wakelee2012Double	11	299	0.037	[0.019; 0.065]
Kim2011Battle	2	98	0.020	[0.002; 0.072]
Chevreau2013Sorafenib	2	13	0.154	[0.019; 0.454]
Dingemans2013Phase	5	57	0.088	[0.029; 0.193]
Papa2013Phase	3	53	0.057	[0.012; 0.157]
Santoro2013Phase	11	76	0.145	[0.075; 0.244]
Savvides2013Phase	2	20	0.100	[0.012; 0.317]
Schwandt2014Randomized	2	13	0.154	[0.019; 0.454]
**Random effects model**		**2693**	**0.074**	**[0.055; 0.099]**
Heterogeneity: I−squared=71%, tau−squared=0.7696, p<0.0001				
phase_meta = p3				
Cheng2009Efficacy	5	149	0.034	[0.011; 0.077]
Escudier2007Sorafenib	44	451	0.098	[0.072; 0.129]
Llovet2008Sorafenib	7	297	0.024	[0.010; 0.048]
Brose2014Sorafenib	24	196	0.122	[0.080; 0.177]
**Random effects model**		**1093**	**0.061**	**[0.031; 0.117]**
Heterogeneity: I−squared=86%, tau−squared=0.4242, p<0.0001				

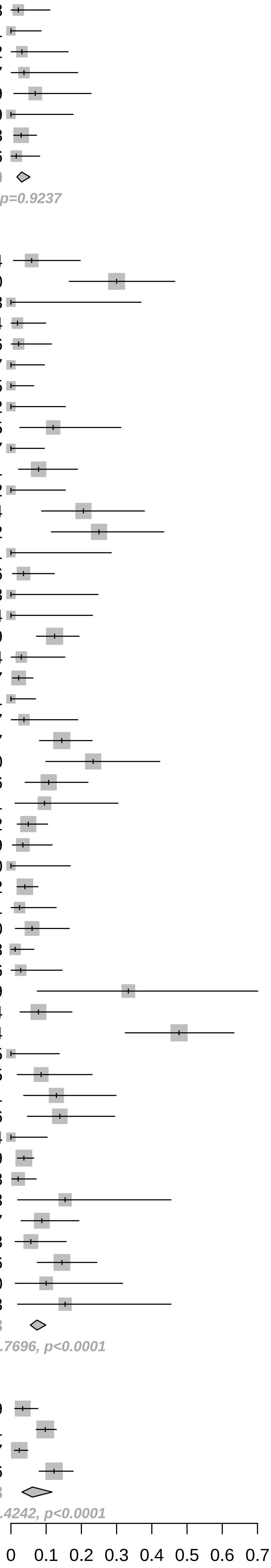

Supplement: S4 Fig — There was no significant difference in objective response rate (p > 0.05) between phase I, phase II and phase III trials. (PDF) [file pbio.2000487.s004.pdf]
