# Supplementary material for: Inefficiencies and Patient Burdens in the Development of the Targeted Cancer Drug Sorafenib: A Systematic Review
Source: PLoS Biol. 2017 Feb 3;15(2):e2000487. doi: 10.1371/journal.pbio.2000487 (PMC5291369; doi:10.1371/journal.pbio.2000487)
Supplement: S2 Table — (DOCX) [file pbio.2000487.s011.docx]

| **First Author, Publication Year** | **Enrolment date** | **Eligibility criteria overlap** | **Eligibility criteria differences** |
| --- | --- | --- | --- |
| Bianchi et al., 2009 [1] | 2004-02-01 | Metastatic breast cancer after prior chemotherapy | Prior chemotherapy for metastatic disease required |
| Moreno-Aspitia et al., 2009 [2] | 2004-11-01 |  | Prior chemotherapy in (neo)adjuvant or metastatic setting with an anthracycline or taxane required |
| Elser et al., 2007 [3] | 2004-01-01 | SCC of Head and Neck | 0-1 prior systemic therapy for metastatic disease |
| Williamson et al., 2010 [4] | 2004-10-01 |  | No prior chemotherapy for metastatic disease, although chemotherapy in (neo)adjuvant setting allowed |
| Dubey et al., 2010 [5] | 2004-10-01 | Malignant mesothelioma | 0-1 pemetrexed-containing chemotherapy regimen |
| Papa et al., 2013 [6] | 2008-11-01 |  | 1 prior pemexetred-platinum therapy |
| Blumenschein et al., 2009 [7] | 2004-04-01 | Advanced, stage 4, previously treated NSCLC | 1-2 prior chemotherapy regimens |
| Kelly et al., 2011 [8] | 2005-01-01 |  | 1+ prior chemotherapy regimens, no prior inhibitors of MEK, FT, RAF, VEGFR |
| Wakelee et al., 2012 [9] | 2004-05-01 | Advanced NSCLC | No stage specified, >=2 prior chemotherapy regimens |
| Kim et al., 2011 [10] | 2006-11-01 |  | Stage 3b/4, >=1 prior chemotherapy regimen |
| Schwandt et al., 2014 [11] | 2004-01-01 | EOC or PPC, prior platinum | Platinum sensitive (no recurrence <6 mo), 1-2 prior cytotoxic regimens |
| Matei et al., 2011 [12] | 2004-10-01 |  | Platinum resistant (recurrence within 12 mo), 1-2 prior chemotherapy regimens |
| Bodnar et al., 2011 [13] | 2008-12-01 |  | Stratified by platinum (enrolled both platinum sensory and refractory patients), 2 prior cytotoxic regimens |
| Safarinejad et al., 2010 [14] | 2004-01-01 | CRPC, 0 prior chemotherapy | Treatment-naïve |
| Chi et al., 2007 [15] | 2004-07-01 |  | Radiation therapy allowed |
| Steinbild et al., 2007 [16] | 2004-08-01 |  | Hormonal therapy allowed |
| Sridhar et al., 2011 [17] | 2005-08-01 | Transitional cell carcinoma of the urothelium | No prior chemotherapy for metastatic disease, although chemotherapy in (neo)adjuvant setting allowed |
| Dreicer et al., 2009 [18] | 2005-10-01 |  | 1 prior systemic therapy for metastatic disease required |
| Kloos et al., 2009 [19] | 2004-10-01 | Metastatic thyroid cancer | No mention of I^131^-refractory patients in eligibility criteria (93% of enrolled patients) |
| Gupta-Abramson et al., 2008 [20] | 2006-02-01 |  | I^131^-refractory patients required (93% of enrolled patients) |
| Srimuninnimit et al., 2014 [21] | 2008-03-01 | Advanced HCC, gemcitabine, no prior systemic treatment | Locoregional therapies allowed |
| Naqi et al., 2014 [22] | 2008-09-01 |  | Treatment-naïve |

**References**

1. Bianchi G, Loibl S, Zamagni C, Salvagni S, Raab G, Siena S, et al. Phase II multicenter, uncontrolled trial of sorafenib in patients with metastatic breast cancer. Anti-cancer drugs. 2009;20(7):616–624.

2. Moreno-Aspitia A, Morton RF, Hillman DW, Lingle WL, Rowland KM, Wiesenfeld M, et al. Phase II trial of sorafenib in patients with metastatic breast cancer previously exposed to anthracyclines or taxanes: North Central Cancer Treatment Group and Mayo Clinic Trial N0336. Journal of Clinical Oncology. 2009;27(1):11–15.

3. Elser C, Siu LL, Winquist E, Agulnik M, Pond GR, Chin SF, et al. Phase II trial of sorafenib in patients with recurrent or metastatic squamous cell carcinoma of the head and neck or nasopharyngeal carcinoma. Journal of Clinical Oncology. 2007;25(24):3766–3773.

4. Williamson SK, Moon J, Huang CH, Guaglianone PP, LeBlanc M, Wolf GT, et al. Phase II evaluation of sorafenib in advanced and metastatic squamous cell carcinoma of the head and neck: Southwest Oncology Group Study S0420. Journal of Clinical Oncology. 2010;28(20):3330–3335.

5. Dubey S, Jänne PA, Krug L, Pang H, Wang X, Heinze R, et al. A phase II study of sorafenib in malignant mesothelioma: results of CALGB 30307. Journal of thoracic oncology: official publication of the International Association for the Study of Lung Cancer. 2010;5(10).

6. Papa S, Popat S, Shah R, Prevost AT, Lal R, McLennan B, et al. Phase 2 study of sorafenib in malignant mesothelioma previously treated with platinum-containing chemotherapy. Journal of Thoracic Oncology. 2013;8(6):783–787.

7. Blumenschein GR, Gatzemeier U, Fossella F, Stewart DJ, Cupit L, Cihon F, et al. Phase II, multicenter, uncontrolled trial of single-agent sorafenib in patients with relapsed or refractory, advanced non–small-cell lung cancer. Journal of Clinical Oncology. 2009;27(26):4274–4280.

8. Kelly RJ, Rajan A, Force J, Lopez-Chavez A, Keen C, Cao L, et al. Evaluation of KRAS Mutations, Angiogenic Biomarkers, and DCE-MRI in Patients with Advanced Non–Small-Cell Lung Cancer Receiving Sorafenib. Clinical Cancer Research. 2011;17(5):1190–1199.

9. Wakelee HA, Lee JW, Hanna NH, Traynor AM, Carbone DP, Schiller JH. A double-blind randomized discontinuation phase II study of sorafenib (BAY 43-9006) in previously treated non-small cell lung cancer patients: Eastern Cooperative Oncology Group study E2501. Journal of thoracic oncology: official publication of the International Association for the Study of Lung Cancer. 2012;7(10):1574.

10. Kim ES, Herbst RS, Wistuba II, Lee JJ, Blumenschein GR, Tsao A, et al. The BATTLE trial: personalizing therapy for lung cancer. Cancer discovery. 2011;1(1):44–53.

11. Schwandt A, von Gruenigen VE, Wenham RM, Frasure H, Eaton S, Fusco N, et al. Randomized phase II trial of sorafenib alone or in combination with carboplatin/paclitaxel in women with recurrent platinum sensitive epithelial ovarian, peritoneal, or fallopian tube cancer. Investigational new drugs. 2014;32(4):729–738.

12. Matei D, Sill MW, Lankes HA, DeGeest K, Bristow RE, Mutch D, et al. Activity of sorafenib in recurrent ovarian cancer and primary peritoneal carcinomatosis: a gynecologic oncology group trial. Journal of Clinical Oncology. 2011;29(1):69–75.

13. Bodnar L, Górnas M, Szczylik C. Sorafenib as a third line therapy in patients with epithelial ovarian cancer or primary peritoneal cancer: a phase II study. Gynecologic oncology. 2011;123(1):33–36.

14. Safarinejad MR. Safety and efficacy of sorafenib in patients with castrate resistant prostate cancer: a phase II study. Urologic Oncology: Seminars and Original Investigations. 2010;28(1):21–27.

15. Chi K, Ellard S, Hotte S, Czaykowski P, Moore M, Ruether J, et al. A phase II study of sorafenib in patients with chemo-naive castration-resistant prostate cancer. Annals of Oncology. 2007;p. mdm554.

16. Steinbild S, Mross K, Frost A, Morant R, Gillessen S, Dittrich C, et al. A clinical phase II study with sorafenib in patients with progressive hormone-refractory prostate cancer: a study of the CESAR Central European Society for Anticancer Drug Research-EWIV. British journal of cancer. 2007;97(11):1480–1485.

17. Sridhar SS, Winquist E, Eisen A, Hotte SJ, McWhirter E, Tannock IF, et al. A phase II trial of sorafenib in first-line metastatic urothelial cancer: a study of the PMH Phase II Consortium. Investigational new drugs. 2011;29(5):1045–1049.

18. Dreicer R, Li H, Stein M, DiPaola R, Eleff M, Roth BJ, et al. Phase 2 trial of sorafenib in patients with advanced urothelial cancer. Cancer. 2009;115(18):4090–4095.

19. Kloos RT, Ringel MD, Knopp MV, Hall NC, King M, Stevens R, et al. Phase II trial of sorafenib in metastatic thyroid cancer. Journal of Clinical Oncology. 2009;27(10):1675–1684.

20. Gupta-Abramson V, Troxel AB, Nellore A, Puttaswamy K, Redlinger M, Ransone K, et al. Phase II trial of sorafenib in advanced thyroid cancer. Journal of Clinical Oncology. 2008;26(29):4714–4719.

21. Srimuninnimit V, Sriuranpong V, Suwanvecho S. Efficacy and safety of sorafenib in combination with gemcitabine in patients with advanced hepatocellular carcinoma: A multicenter, open-label, single-arm phase II study. Asia-Pacific Journal of Clinical Oncology. 2014;10(3):255–260.

22. Naqi N, Ahmad S, Murad S, Khattak J. Efficacy and safety of sorafenib–gemcitabine combination therapy in advanced hepatocellular carcinoma: An open-label Phase II feasibility study. Hematology/oncology and stem cell therapy. 2014;7(1):27–31.
