# Supplementary material for: Inefficiencies and Patient Burdens in the Development of the Targeted Cancer Drug Sorafenib: A Systematic Review
Source: PLoS Biol. 2017 Feb 3;15(2):e2000487. doi: 10.1371/journal.pbio.2000487 (PMC5291369; doi:10.1371/journal.pbio.2000487)
Supplement: S1 Text — (DOCX) [file pbio.2000487.s012.docx]

**Embase**

Database: Embase <1980 to 2014 Week 42.

1. exp "randomized controlled trial"/

2. exp "randomized controlled trial (topic)"/

3. exp "controlled clinical trial"/

4. exp "controlled clinical trial (topic)"/

5. exp randomization/

6. double blind procedure/

7. exp placebo/

8. "controlled clinical trial".tw.

9. (random* or RCT$1 or placebo*).tw.

10. ((singl* or doubl* or trebl* or tripl*) and (mask* or blind* or dumm*)).tw.

11. or/1-10

12. exp clinical trial/

13. "clinical trial".tw.

14. (volunteer or volunteers or open label* or nonrandom* or non random* or quasirandom* or quasi-random*).tw.

15. (longitudinal or prospective).tw.

16. ((follow-up or followup) adj stud*).tw.

17. ((multicenter adj stud*) or (multi-center adj stud*) or (multicentr* adj stud*) or (multi-centr* adj stud*)).tw.

18. ((comparative adj study) or (comparative adj studies)).tw.

19. "head-to-head".tw.

20. (pilot$1 or feasibility or "Proof of principle").tw.

21. or/12-20

22. 11 or 21

23. (editorial or letter or note).pt.

24. 22 not 23

25. exp Animal/ not (exp Animal/ and Human/)

26. 24 not 25

27. (sorafenib or Nexavar or sofafenibum or "BAY 43-9006" or "BAY-43-9006" or "BAY 439006" or "BAY43-9006").tw.

28. 26 and 27

**MEDLINE**

Database: Ovid MEDLINE(R) In-Process & Other Non-Indexed Citations and Ovid MEDLINE(R) <1946 to Present>

1. (controlled clinical trial or randomized controlled trial).pt.

2. exp randomized controlled trials as topic/ or exp controlled clinical trials as topic/ or exp random allocation/ or exp double-blind method/ or exp single-blind method/ or exp placebos/

3. "controlled clinical trial".tw.

4. (random* or RCT$1 or placebo*).tw.

5. ((singl* or doubl* or trebl* or tripl*) and (mask* or blind* or dumm*)).tw.

6. or/1-5

7. clinical trial.pt.

8. (clinical trial phase i or clinical trial phase ii or clinical trial phase iii or clinical trial phase iv).pt.

9. exp Clinical Trial/

10. exp Clinical Trials as Topic/

11. "clinical trial".tw.

12. (volunteer or volunteers or open label* or nonrandom* or non random* or quasirandom* or quasi-random*).tw.

13. exp Longitudinal Studies/ or exp Prospective Studies/ or exp Follow-Up Studies/

14. (longitudinal or prospective).tw.

15. ((follow-up or followup) adj stud*).tw.

16. Multicenter Study.pt.

17. exp Multicenter Study/ or exp Multicenter Studies as Topic/

18. ((multicenter adj stud*) or (multi-center adj stud*) or (multicentr* adj stud*) or (multi-centr* adj stud*)).tw.

19. Comparative Study.pt.

20. ((comparative adj study) or (comparative adj studies)).tw.

21. "head-to-head".tw.

22. exp Pilot Projects/ or exp Feasibility Studies/

23. (pilot$1 or feasibility or "Proof of principle").tw.

24. or/7-23

25. 6 or 24

26. (comment or editorial or guideline or practice guideline or interview or letter).pt.

27. 25 not 26

28. exp Animals/ not (exp Animals/ and Humans/)

29. 27 not 28

30. (sorafenib or Nexavar or sofafenibum or "BAY 43-9006" or "BAY-43-9006" or "BAY 439006" or "BAY43-9006").tw.

31. 29 and 30
