# Supplementary material for: Inefficiencies and Patient Burdens in the Development of the Targeted Cancer Drug Sorafenib: A Systematic Review
Source: PLoS Biol. 2017 Feb 3;15(2):e2000487. doi: 10.1371/journal.pbio.2000487 (PMC5291369; doi:10.1371/journal.pbio.2000487)
Supplement: S2 Text — (DOCX) [file pbio.2000487.s013.docx]

Duplicative trials.[1,2,3,4,5,6,7,8,9,10]

Trials lacking appropriate follow-up.[11,12,13,14,15,6,16,17,18,19,20,9,21,22]

Trials demonstrating accrual failure.[23,24,25,26,27,28,29,30,31,32,33]

References

1. Wörns MA, Weinmann A, Pfingst K, Schulte-Sasse C, Messow CM, Schulze-Bergkamen H, et al. Safety and efficacy of sorafenib in patients with advanced hepatocellular carcinoma in consideration of concomitant stage of liver cirrhosis. Journal of clinical gastroenterology. 2009;43(5):489–495.

2. Pressiani T, Boni C, Rimassa L, Labianca R, Fagiuoli S, Salvagni S, et al. Sorafenib in patients with Child-Pugh class A and B advanced hepatocellular carcinoma: a prospective feasibility analysis. Annals of oncology. 2013;24(2):406–411.

3. Flaherty KT, Lathia C, Frye RF, Schuchter L, Redlinger M, Rosen M, et al. Interaction of sorafenib and cytochrome P450 isoenzymes in patients with advanced melanoma: a phase I/II pharmacokinetic interaction study. Cancer chemotherapy and pharmacology. 2011;68(5):1111–1118.

4. Steinbild S, Mross K, Frost A, Morant R, Gillessen S, Dittrich C, et al. A clinical phase II study with sorafenib in patients with progressive hormone-refractory prostate cancer: a study of the CESAR Central European Society for Anticancer Drug Research-EWIV. British journal of cancer. 2007;97(11):1480–1485.

5. Jonasch E, Corn P, Pagliaro LC, Warneke CL, Johnson MM, Tamboli P, et al. Upfront, randomized, phase 2 trial of sorafenib versus sorafenib and low-dose interferon alfa in patients with advanced renal cell carcinoma. Cancer. 2010;116(1):57–65.

6. Zustovich F, Landi L, Lombardi G, Porta C, Galli L, Fontana A, et al. Sorafenib plus daily low-dose temozolomide for relapsed glioblastoma: a phase II study. Anticancer research. 2013;33(8):3487–3494.

7. Pawlik TM, Reyes DK, Cosgrove D, Kamel IR, Bhagat N, Geschwind JFH. Phase II trial of sorafenib combined with concurrent transarterial chemoembolization with drug-eluting beads for hepatocellular carcinoma. Journal of Clinical Oncology. 2011;29(30):3960–3967.

8. McDermott DF, Sosman JA, Gonzalez R, Hodi FS, Linette GP, Richards J, et al. Double-blind randomized phase II study of the combination of sorafenib and dacarbazine in patients with advanced melanoma: a report from the 11715 Study Group. Journal of Clinical Oncology. 2008;26(13):2178–2185.

9. Lind JS, Dingemans AMC, Groen HJ, Thunnissen FB, Bekers O, Heideman DA, et al. A multicenter phase II study of erlotinib and sorafenib in chemotherapy-naive patients with advanced non–small cell lung cancer. Clinical Cancer Research. 2010;16(11):3078–3087.

10. El-Khoueiry A, Ramanathan R, Yang D, Zhang W, Shibata S, Wright J, et al. A randomized phase II of gemcitabine and sorafenib versus sorafenib alone in patients with metastatic pancreatic cancer. Investigational new drugs. 2012;30(3):1175–1183.

11. Park S, Ryu M, Ryoo B, Im S, Kwon H, Lee S, et al. Sorafenib in patients with metastatic gastrointestinal stromal tumors who failed two or more prior tyrosine kinase inhibitors: a phase II study of Korean gastrointestinal stromal tumors study group. Investigational new drugs. 2012;30(6):2377–2383.

12. Guidetti A, Carlo-Stella C, Locatelli SL, Malorni W, Pierdominici M, Barbati C, et al. Phase II study of sorafenib in patients with relapsed or refractory lymphoma. British journal of haematology. 2012;158(1):108–119.

13. Grignani G, Palmerini E, Dileo P, Asaftei S, D’Ambrosio L, Pignochino Y, et al. A phase II trial of sorafenib in relapsed and unresectable high-grade osteosarcoma after failure of standard multimodal therapy: an Italian Sarcoma Group study. Annals of Oncology. 2011;p. mdr151.

14. Galal K, Khaled Z, Mourad AM, et al. Role of cetuximab and sorafenib in treatment of metastatic colorectal cancer. Indian journal of cancer. 2011;48(1):47.

15. Sun W, Powell M, O’Dwyer PJ, Catalano P, Ansari RH, Benson AB. Phase II study of sorafenib in combination with docetaxel and cisplatin in the treatment of metastatic or advanced gastric and gastroesophageal junction adenocarcinoma: ECOG 5203. Journal of Clinical Oncology. 2010;28(18):2947–2951.

16. Yang Y, Lu Y, Wang C, Bai W, Qu J, Chen Y, et al. Cryotherapy is associated with improved clinical outcomes of sorafenib therapy for advanced hepatocellular carcinoma. Cell biochemistry and biophysics. 2012;63(2):159–169.

17. Xue C, Huang Y, Huang P, Yu Q, Pan J, Liu L, et al. Phase II study of sorafenib in combination with cisplatin and 5-fluorouracil to treat recurrent or metastatic nasopharyngeal carcinoma. Annals of oncology. 2013;24(4):1055–1061.

18. Ravandi F, Alattar ML, Grunwald MR, Rudek MA, Rajkhowa T, Richie MA, et al. Phase 2 study of azacytidine plus sorafenib in patients with acute myeloid leukemia and FLT-3 internal tandem duplication mutation. Blood. 2013;121(23):4655–4662.

19. Amaravadi RK, Schuchter LM, McDermott DF, Kramer A, Giles L, Gramlich K, et al. Phase II trial of temozolomide and sorafenib in advanced melanoma patients with or without brain metastases. Clinical Cancer Research. 2009;15(24):7711–7718.

20. Gridelli C, Morgillo F, Favaretto A, De Marinis F, Chella A, Cerea G, et al. Sorafenib in combination with erlotinib or with gemcitabine in elderly patients with advanced non-small-cell lung cancer: a randomized phase II study. Annals of oncology. 2011;22(7):1528–1534.

21. Maroto J, Del Muro X, Mellado B, Perez-Gracia J, Andrés R, Cruz J, et al. Phase II trial of sequential subcutaneous interleukin-2 plus interferon alpha followed by sorafenib in renal cell carcinoma (RCC). Clinical and Translational Oncology. 2013;15(9):698–704.

22. Bellmunt J, Trigo JM, Calvo E, Carles J, Pérez-Gracia JL, Rubió J, et al. Activity of a multitargeted chemo-switch regimen (sorafenib, gemcitabine, and metronomic capecitabine) in metastatic renal-cell carcinoma: a phase 2 study (SOGUG-02-06). The lancet oncology. 2010;11(4):350–357.

23. von Mehren M, Rankin C, Goldblum JR, Demetri GD, Bramwell V, Ryan CW, et al. Phase 2 Southwest Oncology Group-directed intergroup trial (S0505) of sorafenib in advanced soft tissue sarcomas. Cancer. 2012;118(3):770–776.

24. Petrini I, Lencioni M, Ricasoli M, Iannopollo M, Orlandini C, Oliveri F, et al. Phase II trial of sorafenib in combination with 5-fluorouracil infusion in advanced hepatocellular carcinoma. Cancer chemotherapy and pharmacology. 2012;69(3):773–780.

25. Lam ET, Ringel MD, Kloos RT, Prior TW, Knopp MV, Liang J, et al. Phase II clinical trial of sorafenib in metastatic medullary thyroid cancer. Journal of Clinical Oncology. 2010;28(14):2323–2330.

26. Ott PA, Hamilton A, Min C, Safarzadeh-Amiri S, Goldberg L, Yoon J, et al. A phase II trial of sorafenib in metastatic melanoma with tissue correlates. PLoS One. 2010;5(12):e15588.

27. Martin-Richard M, Gallego R, Pericay C, Foncillas JG, Queralt B, Casado E, et al. Multicenter phase II study of oxaliplatin and sorafenib in advanced gastric adenocarcinoma after failure of cisplatin and fluoropyrimidine treatment. A GEMCAD study. Investigational new drugs. 2013;31(6):1573–1579.

28. Santoro A, Comandone A, Basso U, Parra HS, De Sanctis R, Stroppa E, et al. Phase II prospective study with sorafenib in advanced soft tissue sarcomas after anthracycline-based therapy. Annals of oncology. 2013;24(4):1093–1098.

29. Savvides P, Nagaiah G, Lavertu P, Fu P, Wright JJ, Chapman R, et al. Phase II trial of sorafenib in patients with advanced anaplastic carcinoma of the thyroid. Thyroid. 2013;23(5):600–604.

30. Schwartzberg LS, Tauer KW, Hermann RC, Makari-Judson G, Isaacs C, Beck JT, et al. Sorafenib or Placebo with Either Gemcitabine or Capecitabine in Patients with HER-2–Negative Advanced Breast Cancer That Progressed during or after Bevacizumab. Clinical Cancer Research. 2013;19(10):2745–2754.

31. Krege S, Rexer H, Dorp F, Geeter P, Klotz T, Retz M, et al. Prospective randomized double-blind multicentre phase II study comparing gemcitabine and cisplatin plus sorafenib chemotherapy with gemcitabine and cisplatin plus placebo in locally advanced and/or metastasized urothelial cancer: SUSE (AUO-AB 31/05). BJU international. 2014;113(3):429–436.

32. Mahalingam D, Malik L, Beeram M, Rodon J, Sankhala K, Mita A, et al. Phase II study evaluating the efficacy, safety, and pharmacodynamic correlative study of dual antiangiogenic inhibition using bevacizumab in combination with sorafenib in patients with advanced malignant melanoma. Cancer chemotherapy and pharmacology. 2014;74(1):77–84.

33. Schwandt A, von Gruenigen VE, Wenham RM, Frasure H, Eaton S, Fusco N, et al. Randomized phase II trial of sorafenib alone or in combination with carboplatin/paclitaxel in women with recurrent platinum sensitive epithelial ovarian, peritoneal, or fallopian tube cancer. Investigational new drugs. 2014;32(4):729–738.
