# Supplementary material for: Inefficiencies and Patient Burdens in the Development of the Targeted Cancer Drug Sorafenib: A Systematic Review
Source: PLoS Biol. 2017 Feb 3;15(2):e2000487. doi: 10.1371/journal.pbio.2000487 (PMC5291369; doi:10.1371/journal.pbio.2000487)
Supplement: S3 Text — (DOCX) [file pbio.2000487.s014.docx]

# Extraction

See attached codebook.

# Main summary measures

The main summary measures for an extracted drug are as follows.

- Number of primary publications extracted
- Overall number and stratify by monotherapy and combination therapy
- Number of patients involved
- Number of years from first trial to last
- Number of separate malignancies tested
- Number of combination therapies tested
- Estimate for patient-years of involvement
- Sum of per-trial products of duration of treatment and number of patients
- Overall number and stratify by monotherapy and combination therapy
- Response rate by RECIST criteria
- Numerator is the sum the total number of partial and complete responses for the arm of interest in the trial
- Denominator is the number of patients in the intent-to-treat subgroup
- Pooled proportion of responses: using the *metaprop* function in the *meta* package for R, calculate a weighted estimate for the pooled proportion of responses using a random effects model
- Report the sum of the number of responses; pooled proportion estimate and 95% confidence intervals
- Grade 3-4 severe adverse event (SAE) rate by CTCAE criteria
- Numerator is the sum of grade 3-4 SAE's in the arm of interest
- Denominator is the number of patients included in the safety analysis
- Pooled proportion of events: using the *metaprop* function in the *meta* package for R, calculate a weighted estimate for the pooled SAE rate using a random effects model
- Report the sum of the number of SAE's; pooled proportion estimate and 95% confidence intervals
- Grade 5 SAE (death) rate
- Numerator is the sum of deaths stated by the investigator to be at least “possibly” or “probably” treatment-related in the arm of interest
- Denominator is the number of patients included in the safety analysis
- Pooled proportion of events: using the *metaprop* function in the *meta* package for R, calculate a weighted estimate for the pooled SAE rate using a random effects model
- Report the sum of the number of deaths; pooled proportion estimate and 95% confidence intervals

For all measures, only consider responses and events that are in an arm of a study that is considering the drug of interest. E.g. for a sorafenib extraction, consider only the sorafenib arm and exclude the responses and events from the placebo arm.

# Trials of marginal value

A trial will be considered to be of “marginal value” if it satisfies one of the following three conditions:

- Potentially underpowered: The trial has not accrued at least 85% of its expected enrolment
- Duplicative: The trial was initiated after the publication of another trial in the same indication that failed to meet its primary endpoint
- Unredeemed signal: A phase 2 met its primary efficacy endpoint, had an acceptable toxicity profile, was recommended for phase 3 testing, but was never followed up with a phase 3 trial

The following will be calculated for trials of marginal value.

- Number of patients involved
- Number of patient-years of involvement
- Number of responses, grade 3-4 SAE's, deaths
- Pooled proportion of responses and events: using the *metaprop* function in the *meta* package for R, calculate a weighted estimate for the pooled response and SAE rate using a random effects model
- Report proportion estimates and 95% confidence intervals
- Report the percent of SAE's and patient-years of involvement in trials of marginal value in the total portfolio
