# Supplementary material for: Inefficiencies and Patient Burdens in the Development of the Targeted Cancer Drug Sorafenib: A Systematic Review
Source: PLoS Biol. 2017 Feb 3;15(2):e2000487. doi: 10.1371/journal.pbio.2000487 (PMC5291369; doi:10.1371/journal.pbio.2000487)
Supplement: S4 Text — (DOCX) [file pbio.2000487.s015.docx]

# A. Basic information

- Location of corresponding author
- Registration number
  - From www.clinicaltrials.gov, if given in the text only.
- Publication date
  - Earliest publication date, including e-publication, if available.
- Stated goal: Safety, Dosage, Efficacy, Pharmacodynamics, Pharmacokinetics, Other
  - All articulated goals, anywhere in the manuscript. Often will occur in methods or intro as a list of primary and secondary endpoints. Avoid imputing goals.
  - Do not put safety, unless safety is explicitly mentioned as a goal before the results section.
- Sponsor: Government, Biotech, Pharma, Nonprofit, Not stated
  - Provided any funding to support study. Criteria for sponsorship: named explicitly as supplying funding in article.
  - Criteria for "Biotech:" Involved primarily in discovery as opposed to manufacture, or manufactures products primarily using biological processes. Some biotech companies are owned by big pharma, but ownership is irrelevant for classification. Hence, some common biotech firms include: Amgen, Biogen, Genentech, Genzyme, etc.
  - Pharma includes Merck, Pfizer, etc. When there is doubt, add to directory of companies.
  - Criteria for dividing ambiguous entity should be whether main revenue stream is a biotech derived product (e.g. recombinant protein) or a small molecule. "Develops and markets" means that it's a pharma.
- What is the phase of the trial? Case, Phase 1, Phase 1-2, Phase 2, Phase 2-3, Phase 3
  - If the phase number is not explicitly stated:
  - Phase 1: healthy volunteers OR patients lacking target disorder, OR dose escalation where safety, dosage, OR PK are primary endpoints. Typically < 50 patients.
  - Phase 2: in patients with target disorder, AND primary endpoint not specified OR primary endpoint is specified, and it is a surrogate (i.e. tumor response), ... other pieces of evidence: call for large randomized trials
  - Phase 3: "confirmatory," "pivotal", OR randomized trial enrolling > 200 patients where primary endpoint is clearly specified, and it is a clinical endpoint. Typically uses 1 or perhaps 2 dose arms.
- Is the phase of the trial stated in the paper itself? Phase is explicitly stated, Phase is not stated
- Single or multi centre?
- What is the indication for the trial?
  - Short phrase capturing broad clinical indication. Do not include criteria for classification. E.g. "RCC," "partial onset seizures," "Parkinson's." For cancer, indication might be "NSCLC."
- Subindication
  - Subset or clinical characteristic of disease, like "Recent onset Parkinson's" or "familial PD." For cancer, subindication might be "EGFR positive NSCLC."
- Stage: First line, Second line, Refractory, Mixed
  - 1st line: no prior therapy
  - 2nd line: refractory to / relapsed after at least one prior therapy
  - Refractory: "inadequately controlled," "advanced," or "metastatic"
- Are investigator conflicts of interest disclosed?
  - Discloses anywhere in the manuscript the existence or nonexistence of a financial relationship with sponsor.
- Is there a conflict of interest?
  - If yes, this means that there is a significant relationship among any of authors and sponsor. All conflicts of interest count, including funding, consultancies, etc.
  - "Supported by Pfizer" would not be a conflict of interest. This only includes relationships between the sponsor and investigators.

# B. Rationale for investigation

- Introduction citations
  - This section records the stated evidentiary rationale supporting the clinical trial at hand, based on references supplied by the author. We are not seeking citations to articles on chemistry, manufacturing, etc. Rather, grounds for believing drug will modulate particular effect.
  - Citations: list each citation of experimental evidence that seems to include live animals (preclinical or clinical) used to support the trial. We will later code this according to evidence type.
  - Begin by typing an author's name or part of the title of the citation. When you have found the citation you're looking for, click "Add citation" and this will attach it to this extraction. If you can't find the citation in the list of suggestions that appears, choose "Add a new reference" from the panel at the left.
  - Indication in the text: Look at the sentence preceding the citation, and record indications that are referred to in that sentence. Don't go looking at the article being cited.
  - Efficacy / safety / dosage: Indicate why the article is being cited.
  - Cited as positive (P / N / I): Indicate if the article is cited as positive, negative or inconclusive.

# C. Methods

## 1. Hypothesis

- Randomization: Randomized, Not randomized
  - With respect to the drug in question
- Primary outcome blinding
- Allocation blinding
- Description of withdrawals
  - Withdrawals described: any description of withdrawals
  - Withdrawals not described: no description of withdrawals at all

## 2. Design

- Design: Dose escalation, Dose finding, Dose ranging, Historical control, Parallel group, Futility, Simon two-stage, Placebo, Crossover, Invasv PD/BM, Other (specify)
  - Dose finding: mainly to cause side-effects, titrates dose to pre-specified optimum based on bio or clinical specifications.
  - Dose ranging: tests specified doses.
  - Dose escalation: determines doses for subsequent cohorts.

## 3. Treatment

- Experimental arms
  - N: number of patients at baseline. Do not record patients screened. Typically, this is number randomised, though if there is a 2 week wash-out before randomization, N would be recorded at start of wash-out.
  - Not the number of patients assessable or the number enrolled, generally the number that started the treatment.
  - D: day. W: week, M: month, X: cycle
  - Ex: "Pts received DRUG 14 mg/m2 as a 4-hour intravenous infusion on days 1, 8, and 15 of each 28-day cycle for up to six cycles" would be coded as: "D 1, 8, 15 / 28 d X 6"
  - If "combination" is selected, please indicate the other drug being used in the combination therapy. Leave blank if none.
  - Route: Oral, IV, Surgical, Other

| Arm (e.g. sorafenib or placebo) | Arm N | Dose | Schedule | Route | Combination |
| --- | --- | --- | --- | --- | --- |
|  |  |  |  |  |  |
|  |  |  |  |  |  |
|  |  |  |  |  |  |

- Outcomes
  - Only put "safety" if it is the primary endpoint
  - Priority: 1 for primary outcome, 2 for secondary outcome(s)

| Outcome | Priority |
| --- | --- |
|  |  |
|  |  |
|  |  |

## 4. Time

- Duration of patient enrolment from baseline to primary endpoint (weeks)
  - Indicate how long a patient must be enrolled in a trial in order for the primary endpoint to be collected. Captures length of time between projected collection of primary endpoint and collection of baseline data.
  - If trial is "time to event" (i.e. indeterminate endpoint) use mean or median time to event for overall study.
  - Something like PFS-6 would be recorded as "6 months." (Convert to weeks.)
  - If not specified, impute by median number of cycles x period of each cycle. If total number of cycles given, divide by number of patients, and multiply by period for each cycle. Round to nearest day (i.e. if trial only runs for 6 hours, round to 1 day).
  - If total cycles are given, divide by number of patients and multiply by weeks per cycle.
  - Always report in weeks. If given in months, divide by 12 and multiply by 52.
- Is the duration of treatment imputed?
- Date of enrolment
  - Year and month when first patient was enrolled. If not available, check www.clinicaltrials.gov. Otherwise leave blank and query investigators later.
- Date of closure
  - Year and month when last patient was enrolled. Clinicaltrials.gov does not generally record this info. As above, leave blank if not available.

## 5. Population

- Concomitant medications for managing target disorder?
  - Experimental intervention is added on on top of standard treatment for managing disorder. Do not record meds for managing symptoms of disorder (e.g. nausea for a cancer drug), or comorbidities (e.g. high blood pressure in stroke victims). Do not record brief challenge removal of concomitant (gets recorded in wash-out below).
- Paediatric subjects: All, Some, None
  - Pediatric Subjects: Age < 18 yrs
- Burdens: Invasive research procedures, Wash-out, Other, None

# D. Results

- Primary endpoint: Negative, Inconclusive trend negative, Inconclusive, Inconclusive trend positive, Positive
  - If possible, measure 95% confidence intervals as relate to null hypothesis. Often inferable from text, but may require analysis later on.
  - Negative: upper bound of CI does not capture null
  - Inconclusive trend negative: favours comparator, but not significant
  - Inconclusive: equivalent activity
  - Inconclusive trend positive: favours experimental drug, but not significant
  - Positive: favours new drug and statistically significant
- Toxicity described as: Acceptable, Unacceptable, Not stated
- Total assessment of risk / benefit: Favourable, Inconclusive, Unfavourable
  - Based on last paragraph of paper
- What is the number of patients to receive the drug in question? (Denominator for response)
  - For example, if you are extracting Sorafenib trials, you would put the total number of patients to receive Sorafenib.
  - In cases of single-arm studies, this number may be the same as the total N, below.
  - In cases of placebo control studies, this may be the same number as the arm N for Sorafenib above.
  - This will differ from the arm N above in cases where there is more than one arm receiving the drug in question. Imagine a study where one arm is testing continuous daily dosing and the other is testing the 4/2 schedule. In this case, you would enter the sum of both.
  - This may differ from the total N below, which is the total number enrolled, where here we're extracting the total number receiving the drug in question
- What is the total N?
  - This is the total number of patient-subjects enrolled. The "arm n" from the table above is the total number randomised and treated.
- Efficacy endpoints table
  - Outcome: record outcome measure used. Usually these will be efficacy measures like response rate or survival. However, occasionally, "safety" is a primary or secondary endpoint, and analyses of safety are different than what is captured in Safety Table. When this occurs, record safety info in this table (i.e. for a drug known to cause headaches, and where a primary or secondary outcome is measure of headache events, record average number of headaches). If resp is measured by > 2 methods (i.e. investigator and independent committee assessment), pick only the best standard [independent committee].
  - Arm: which intervention arm? For 2 arm studies, put "Tx" and "control." Record nature of each arm if > 2 (i.e. dose arm)
  - Total N: number of patients at baseline. Do not record patients screened. Typically, this is number randomised, though if there is a 2 week wash-out before randomization, N would be recorded at start of wash-out.
  - N: number in each arm. Rules as above.
  - Mean: record value at specified endpoint in trial. This might be an average measure (-4.3 on UPDRS scale). Or it might be a number of pts (5 objective responses).
  - Aggregated: measures that combine results in treatment and comparator arms. These include: odds ratios, etc.
  - Dir: direction with respect to experimental drug
  - + favors experimental drug
  - 0 equivalent
  - - disfavors experimental drug
  - Sig: statistical tests run
  - + significant
  - 0 NS
  - - insiginficant

| Outcome | Arm | Treated mean value | Stats | Agg. Value | Agg. Var | Agg. Units | Dir | Sig |
| --- | --- | --- | --- | --- | --- | --- | --- | --- |
|  |  |  |  |  |  |  |  |  |
|  |  |  |  |  |  |  |  |  |
|  |  |  |  |  |  |  |  |  |

- What is the statistical measure used to report error? Standard deviation, *p*-value, 95% confidence interval, Standard error, None
- Safety endpoints
  - Probably or definitely treatment related events only
  - SAE: serious adverse events. Grade 3 or 4 adverse events recorded as SAE. Record highest number of SAE in a single category as reported. Look for per-event table. Find highest G3 or G4 SAE row, record the number of events. (E.g. 30 G3 or G4 neutropenia events, record "30".) Do not include deaths.
  - Withdrawals: Withdrawal or discontinuation due to toxicity

| Arm | Deaths | Withdrawals | Grade 3-5 SAE |
| --- | --- | --- | --- |
|  |  |  |  |
|  |  |  |  |
|  |  |  |  |

# E. Discussion

- Does the paper conclude that the drug is of clinical interest? Concludes drug is of clinical interest, Concludes drug is not of clinical interest, Concludes drug should be re-tested, Unclear
- Discussion citations
  - Include case reports and all studies with live animals, preclinical or clinical.
  - Not negative: "Our study compares favourably."
  - Negative: "This study showed toxicities or lack of efficacy."
  - Leave "Cited as negative?" blank if it is not cited as negative.
